# Supplementary material for: Ethanolic extract of Ya-nang (Tiliacora triandra) leaf powder induces apoptosis in cholangiocarcinoma cell lines via induction of hyperacetylation and inhibition of growth signaling
Source: PeerJ. 2022 Dec 15;10:e14518. doi: 10.7717/peerj.14518 (PMC9760018; doi:10.7717/peerj.14518)
Supplement: Supplemental Information 1 [file peerj-10-14518-s001.docx]

**Table S1.** The absorbance values showed cells viability of KKU-M213B, KKU-100 and H69 cells by a microplate reader in duplicate.

| **Cells** |  | **Sample (µl/ml)** | **Exp.1** | | | | | | **Exp.2** | | | | | |
| --- | --- | --- | --- | --- | --- | --- | --- | --- | --- | --- | --- | --- | --- | --- |
|  |  |  | **A550-A655 (1)** | | | **A550-A655 (2)** | | | **A550-A655 (1)** | | | **A550-A655 (2)** | | |
|  |  |  | **1** | **2** | **3** | **1** | **2** | **3** | **1** | **2** | **3** | **1** | **2** | **3** |
| **KKU-M213B** | **24h** | **Control** | 1.20 | 1.24 | 1.34 | 1.31 | 1.36 | 1.44 | 1.21 | 1.18 | 1.23 | 1.45 | 1.47 | 1.54 |
|  |  | **0** | 1.14 | 1.24 | 1.18 | 1.25 | 1.38 | 1.29 | 1.04 | 1.14 | 1.10 | 1.33 | 1.39 | 1.40 |
|  |  | **31.25** | 1.06 | 1.26 | 0.75 | 1.19 | 1.34 | 0.83 | 0.96 | 0.71 | 0.70 | 1.28 | 0.86 | 0.88 |
|  |  | **62.50** | 0.71 | 0.71 | 0.71 | 0.78 | 0.78 | 0.79 | 0.65 | 0.72 | 0.80 | 0.77 | 0.87 | 0.96 |
|  |  | **125** | 0.37 | 0.36 | 0.36 | 0.41 | 0.39 | 0.38 | 0.38 | 0.49 | 0.50 | 0.41 | 0.48 | 0.59 |
|  |  | **250** | 0.31 | 0.28 | 0.33 | 0.31 | 0.27 | 0.34 | 0.43 | 0.46 | 0.45 | 0.47 | 0.46 | 0.49 |
|  |  | **500** | 0.08 | 0.05 | 0.19 | 0.08 | 0.04 | 0.18 | 0.11 | 0.12 | 0.12 | 0.11 | 0.10 | 0.10 |
|  | **48h** | **Control** | 1.72 | 1.85 | 1.89 | 1.76 | 1.88 | 1.92 | 1.86 | 1.82 | 1.83 | 1.88 | 1.85 | 1.85 |
|  |  | **0** | 1.72 | 1.76 | 1.74 | 1.75 | 1.78 | 1.77 | 1.75 | 1.75 | 1.77 | 1.79 | 1.80 | 1.80 |
|  |  | **3.75** | 1.66 | 1.84 | 1.56 | 1.69 | 1.86 | 1.58 | 1.58 | 1.40 | 1.49 | 1.62 | 1.44 | 1.52 |
|  |  | **7.5** | 1.28 | 1.50 | 0.82 | 1.31 | 1.53 | 0.83 | 1.09 | 1.25 | 1.14 | 1.11 | 1.28 | 1.16 |
|  |  | **15** | 0.03 | 0.04 | 0.02 | 0.03 | 0.04 | 0.02 | 0.02 | 0.02 | 0.02 | 0.02 | 0.02 | 0.02 |
|  |  | **30** | 0.03 | 0.03 | 0.03 | 0.03 | 0.03 | 0.03 | 0.02 | 0.02 | 0.02 | 0.02 | 0.02 | 0.02 |
|  |  | **Control** | 1.29 | 1.32 | 1.26 | 1.30 | 1.32 | 1.28 | 1.74 | 1.94 | 1.75 | 1.76 | 1.96 | 1.77 |
|  |  | **0** | 1.55 | 1.50 | 1.53 | 1.57 | 1.52 | 1.54 | 1.73 | 1.85 | 1.79 | 1.76 | 1.87 | 1.81 |
|  |  | **9** | 0.80 | 0.73 | 0.73 | 0.81 | 0.74 | 0.74 | 1.14 | 1.13 | 1.05 | 1.14 | 1.14 | 1.06 |
|  |  | **12** | 0.03 | 0.03 | 0.03 | 0.03 | 0.03 | 0.03 | 0.06 | 0.03 | 0.03 | 0.06 | 0.03 | 0.03 |
|  |  | **Control** | 1.61 | 1.55 | 1.56 | 1.64 | 1.57 | 1.59 | 1.55 | 1.59 | 1.56 | 1.57 | 1.62 | 1.60 |
|  |  | **0** | 1.57 | 1.48 | 1.56 | 1.59 | 1.50 | 1.58 | 1.63 | 1.60 | 1.56 | 1.67 | 1.64 | 1.59 |
|  |  | **10** | 0.02 | 0.03 | 0.03 | 0.02 | 0.03 | 0.03 | 0.04 | 0.03 | 0.03 | 0.04 | 0.03 | 0.03 |
|  | **72h** | **Control** | 2.26 | 2.26 | 2.34 | 2.31 | 2.29 | 2.37 | 2.36 | 2.20 | 2.21 | 2.40 | 2.25 | 2.24 |
|  |  | **0** | 2.24 | 2.28 | 2.25 | 2.24 | 2.31 | 2.28 | 1.97 | 2.17 | 2.07 | 2.03 | 2.21 | 2.12 |
|  |  | **3.75** | 1.87 | 1.66 | 1.88 | 1.91 | 1.71 | 1.93 | 1.87 | 1.86 | 1.92 | 1.94 | 1.92 | 1.96 |
|  |  | **7.5** | 1.22 | 1.23 | 1.57 | 1.24 | 1.25 | 1.61 | 1.97 | 1.52 | 0.39 | 2.02 | 1.52 | 0.39 |
|  |  | **15** | 0.03 | 0.04 | 0.03 | 0.03 | 0.04 | 0.03 | 0.03 | 0.03 | 0.02 | 0.03 | 0.03 | 0.02 |
|  |  | **30** | 0.03 | 0.03 | 0.03 | 0.03 | 0.02 | 0.02 | 0.02 | 0.02 | 0.02 | 0.02 | 0.02 | 0.02 |
|  |  | **Control** | 2.61 | 2.58 | 2.11 | 2.55 | 2.58 | 2.12 | 2.70 | 2.79 | 2.79 | 2.70 | 2.80 | 2.79 |
|  |  | **0** | 2.65 | 2.36 | 2.51 | 2.66 | 2.35 | 2.51 | 2.61 | 2.82 | 2.77 | 2.60 | 2.82 | 2.77 |
|  |  | **9** | 0.66 | 0.47 | 0.57 | 0.66 | 0.47 | 0.56 | 0.19 | 0.25 | 0.14 | 0.19 | 0.25 | 0.14 |
|  |  | **12** | 0.02 | 0.02 | 0.02 | 0.02 | 0.02 | 0.02 | 0.02 | 0.02 | 0.03 | 0.03 | 0.02 | 0.03 |
|  |  | **Control** | 2.02 | 2.03 | 2.09 | 2.06 | 2.08 | 2.14 | 2.14 | 2.18 | 2.06 | 2.19 | 2.24 | 2.11 |
|  |  | **0** | 1.98 | 1.91 | 2.05 | 2.01 | 1.93 | 2.08 | 2.06 | 2.19 | 1.95 | 2.10 | 2.22 | 1.99 |
|  |  | **10** | 0.02 | 0.02 | 0.02 | 0.02 | 0.02 | 0.02 | 0.03 | 0.02 | 0.02 | 0.03 | 0.02 | 0.02 |
| **KKU-100** | **24h** | **Control** | 1.09 | 1.11 | 1.07 | 1.13 | 1.15 | 1.10 | 1.01 | 0.99 | 0.98 | 1.04 | 1.02 | 1.01 |
|  |  | **0** | 1.18 | 1.06 | 1.02 | 1.21 | 1.12 | 1.08 | 1.17 | 0.92 | 1.23 | 1.22 | 0.96 | 1.26 |
|  |  | **31.25** | 0.83 | 1.01 | 0.99 | 0.86 | 1.06 | 1.04 | 1.03 | 1.06 | 0.89 | 1.08 | 1.12 | 0.93 |
|  |  | **62.50** | 0.63 | 0.63 | 0.63 | 0.67 | 0.66 | 0.65 | 0.68 | 0.65 | 0.67 | 0.70 | 0.69 | 0.70 |
|  |  | **125** | 0.54 | 0.53 | 0.55 | 0.57 | 0.56 | 0.58 | 0.50 | 0.51 | 0.51 | 0.53 | 0.54 | 0.53 |
|  |  | **250** | 0.52 | 0.50 | 0.47 | 0.55 | 0.52 | 0.50 | 0.50 | 0.49 | 0.49 | 0.52 | 0.52 | 0.51 |
|  |  | **500** | 0.04 | 0.04 | 0.04 | 0.04 | 0.04 | 0.04 | 0.04 | 0.05 | 0.04 | 0.04 | 0.05 | 0.04 |
|  | **48h** | **control** | 1.42 | 1.46 | 1.53 | 1.47 | 1.50 | 1.55 | 1.48 | 1.48 | 1.57 | 1.52 | 1.54 | 1.63 |
|  |  | **0** | 1.36 | 1.37 | 1.40 | 1.42 | 1.41 | 1.44 | 1.47 | 1.45 | 1.44 | 1.50 | 1.48 | 1.48 |
|  |  | **3.25** | 1.25 | 1.30 | 1.35 | 1.31 | 1.34 | 1.38 | 1.36 | 1.44 | 1.32 | 1.38 | 1.45 | 1.38 |
|  |  | **Sample (µl/ml)** | **Exp.1** | | | | | | **Exp.2** | | | | | |
|  |  |  | **A550-A655 (1)** | | | **A550-A655 (2)** | | | **A550-A655 (1)** | | | **A550-A655 (2)** | | |
|  |  |  | **1** | **2** | **3** | **1** | **2** | **3** | **1** | **2** | **3** | **1** | **2** | **3** |
|  |  | **7.5** | 1.17 | 1.42 | 1.19 | 1.22 | 1.39 | 1.20 | 1.41 | 1.33 | 1.30 | 1.41 | 1.34 | 1.31 |
|  |  | **9** | 1.25 | 1.26 | 1.25 | 1.29 | 1.27 | 1.28 | 1.42 | 1.23 | 1.41 | 1.41 | 1.21 | 1.48 |
|  |  | **10** | 1.28 | 1.09 | 1.18 | 1.33 | 1.07 | 0.64 | 1.36 | 1.34 | 1.38 | 1.39 | 1.34 | 1.43 |
|  |  | **12** | 0.93 | 0.60 | 0.76 | 0.94 | 0.58 | 0.79 | 0.83 | 1.22 | 1.00 | 0.83 | 1.10 | 1.04 |
|  |  | **15** | 0.49 | 0.32 | 0.56 | 0.50 | 0.31 | 0.58 | 0.53 | 0.71 | 0.87 | 0.53 | 0.69 | 0.90 |
|  |  | **30** | 0.02 | 0.09 | 0.33 | 0.02 | 0.09 | 0.34 | 0.14 | 0.09 | 0.19 | 0.15 | 0.09 | 0.19 |
|  | **72h** | **control** | 1.69 | 1.80 | 1.74 | 1.75 | 1.82 | 1.79 | 1.79 | 1.68 | 1.57 | 1.78 | 1.70 | 1.62 |
|  |  | **0** | 1.87 | 1.53 | 1.70 | 1.70 | 1.45 | 1.58 | 1.32 | 1.46 | 1.39 | 1.32 | 1.42 | 1.22 |
|  |  | **3.25** | 1.73 | 1.79 | 1.68 | 1.68 | 1.79 | 1.58 | 1.38 | 1.42 | 1.35 | 1.45 | 1.46 | 1.44 |
|  |  | **7.5** | 1.09 | 1.04 | 1.07 | 1.10 | 0.98 | 1.04 | 0.95 | 0.99 | 0.91 | 0.94 | 0.95 | 0.92 |
|  |  | **9** | 0.65 | 0.68 | 0.67 | 0.70 | 0.63 | 0.64 | 0.65 | 0.64 | 0.63 | 0.62 | 0.61 | 0.61 |
|  |  | **10** | 0.59 | 0.34 | 0.34 | 0.61 | 0.33 | 0.35 | 0.20 | 0.46 | 0.26 | 0.20 | 0.46 | 0.27 |
|  |  | **12** | 0.21 | 0.05 | 0.13 | 0.21 | 0.05 | 0.14 | 0.05 | 0.04 | 0.06 | 0.05 | 0.04 | 0.06 |
|  |  | **15** | 0.08 | 0.08 | 0.06 | 0.09 | 0.08 | 0.06 | 0.06 | 0.03 | 0.03 | 0.06 | 0.03 | 0.03 |
|  |  | **30** | 0.02 | 0.03 | 0.03 | 0.03 | 0.03 | 0.03 | 0.02 | 0.03 | 0.02 | 0.02 | 0.03 | 0.02 |
| **H69** | **24h** | **Control** | 1.37 | 1.32 | 1.21 | 1.42 | 1.38 | 1.28 | 1.07 | 1.31 | 1.22 | 1.07 | 1.31 | 1.22 |
|  |  | **0** | 1.24 | 1.24 | 1.21 | 1.32 | 1.26 | 1.26 | 1.01 | 1.38 | 1.38 | 1.01 | 1.38 | 1.38 |
|  |  | **31.25** | 1.12 | 1.10 | 1.12 | 1.19 | 1.14 | 1.17 | 1.07 | 1.07 | 1.14 | 1.06 | 1.07 | 1.14 |
|  |  | **62.50** | 0.92 | 0.96 | 0.98 | 0.95 | 0.98 | 1.01 | 0.96 | 0.98 | 1.02 | 0.96 | 0.98 | 1.03 |
|  |  | **125** | 0.65 | 0.68 | 0.62 | 0.68 | 0.69 | 0.64 | 0.67 | 0.68 | 0.68 | 0.67 | 0.68 | 0.69 |
|  |  | **250** | 0.35 | 0.34 | 0.35 | 0.35 | 0.35 | 0.35 | 0.41 | 0.37 | 0.48 | 0.41 | 0.37 | 0.48 |
|  |  | **500** | 0.08 | 0.16 | 0.23 | 0.11 | 0.16 | 0.23 | 0.28 | 0.24 | 0.26 | 0.28 | 0.25 | 0.26 |
|  | **48h** | **control** | 1.76 | 1.84 | 1.92 | 1.85 | 1.86 | 1.88 | 1.82 | 1.88 | 1.71 | 1.87 | 1.98 | 1.77 |
|  |  | **0** | 1.61 | 1.69 | 1.78 | 1.63 | 1.77 | 1.81 | 1.74 | 1.71 | 1.64 | 1.69 | 1.72 | 1.77 |
|  |  | **3.25** | 1.07 | 1.12 | 1.09 | 1.12 | 1.17 | 1.15 | 1.20 | 1.17 | 1.23 | 1.26 | 1.20 | 1.31 |
|  |  | **7.5** | 1.16 | 1.20 | 1.23 | 1.15 | 1.20 | 1.11 | 0.93 | 1.13 | 1.03 | 0.88 | 1.14 | 1.01 |
|  |  | **9** | 0.91 | 1.13 | 1.09 | 0.99 | 1.16 | 1.06 | 0.81 | 1.01 | 1.18 | 0.83 | 1.00 | 1.22 |
|  |  | **10** | 0.96 | 0.93 | 1.01 | 0.98 | 0.98 | 0.98 | 0.73 | 0.89 | 1.20 | 0.75 | 0.88 | 1.25 |
|  |  | **12** | 0.95 | 0.94 | 0.95 | 0.97 | 0.89 | 0.93 | 0.83 | 1.07 | 0.95 | 0.77 | 1.10 | 0.94 |
|  |  | **15** | 1.03 | 0.92 | 0.68 | 1.06 | 0.96 | 0.68 | 0.74 | 0.80 | 0.76 | 0.71 | 0.81 | 0.87 |
|  |  | **30** | 0.46 | 0.35 | 0.28 | 0.51 | 0.36 | 0.28 | 0.37 | 0.41 | 0.40 | 0.38 | 0.42 | 0.41 |
|  | **72h** | **control** | 1.91 | 1.98 | 2.05 | 2.03 | 2.09 | 2.15 | 1.99 | 2.02 | 1.84 | 2.19 | 2.22 | 2.03 |
|  |  | **0** | 1.79 | 1.89 | 1.90 | 1.99 | 2.04 | 2.03 | 2.16 | 1.90 | 1.94 | 2.01 | 2.15 | 2.04 |
|  |  | **3.25** | 1.60 | 1.49 | 1.63 | 1.62 | 1.55 | 1.61 | 1.67 | 1.42 | 1.24 | 1.61 | 1.44 | 1.38 |
|  |  | **7.5** | 1.51 | 1.30 | 1.41 | 1.59 | 1.40 | 1.50 | 1.22 | 1.33 | 1.23 | 1.23 | 1.39 | 1.15 |
|  |  | **9** | 1.21 | 1.50 | 1.35 | 1.27 | 1.40 | 1.33 | 1.13 | 1.19 | 1.16 | 1.10 | 1.18 | 1.19 |
|  |  | **10** | 1.27 | 1.31 | 1.42 | 1.36 | 1.26 | 1.34 | 1.12 | 1.14 | 1.17 | 1.16 | 1.20 | 1.15 |
|  |  | **12** | 1.18 | 0.94 | 0.88 | 1.17 | 0.95 | 0.86 | 0.91 | 0.91 | 1.18 | 0.88 | 0.89 | 1.06 |
|  |  | **15** | 0.97 | 0.78 | 0.59 | 1.05 | 0.77 | 0.64 | 0.59 | 0.74 | 0.89 | 0.64 | 0.80 | 0.83 |
|  |  | **30** | 0.24 | 0.21 | 0.29 | 0.25 | 0.22 | 0.28 | 0.23 | 0.28 | 0.20 | 0.23 | 0.29 | 0.21 |

**Table S2.** The percentages of cells were presented the population of each cell cycle phases of KKU-M213B, KKU-100 and H69 cells. Cells were stained with propidium iodide (PI) and analyzed by flow cytometry in duplicate.

| **Cells** | **Sample (µg/ml)** | **Cell cycle phases (%)** | | | | | | | |
| --- | --- | --- | --- | --- | --- | --- | --- | --- | --- |
|  |  | **SubG1** | | **G0/G1** | | **S** | | **G2/M** | |
|  |  | **1** | **2** | **1** | **2** | **1** | **2** | **1** | **2** |
| **KKU-M213B** | **Solvent control** | 6.00 | 7.20 | 78.60 | 78.60 | 4.80 | 4.20 | 10.60 | 10.00 |
|  | **31.25** | 14.00 | 10.50 | 77.10 | 77.70 | 4.10 | 4.70 | 4.70 | 7.00 |
|  | **62.50** | 16.00 | 18.10 | 77.20 | 77.30 | 3.20 | 2.60 | 3.40 | 2.00 |
|  | **125.00** | 20.00 | 19.30 | 69.50 | 66.30 | 4.80 | 6.10 | 5.20 | 8.30 |
| **KKU-100** | **Solvent control** | 1.00 | 0.60 | 70.40 | 73.60 | 11.30 | 13.10 | 16.70 | 12.10 |
|  | **31.25** | 1.10 | 1.50 | 83.60 | 82.20 | 7.20 | 8.60 | 7.80 | 7.20 |
|  | **62.50** | 1.60 | 1.70 | 84.30 | 84.10 | 6.40 | 6.40 | 7.50 | 7.40 |
|  | **125.00** | 7.30 | 7.30 | 84.60 | 84.50 | 4.20 | 3.70 | 3.80 | 4.60 |
| **H69** | **Solvent control** | 3.90 | 3.50 | 43.70 | 48.80 | 19.20 | 10.50 | 26.00 | 37.20 |
|  | **31.25** | 8.40 | 6.30 | 46.20 | 50.60 | 18.70 | 11.30 | 22.00 | 31.80 |
|  | **62.50** | 8.70 | 8.30 | 50.10 | 52.90 | 18.10 | 10.90 | 19.50 | 27.90 |
|  | **125** | 9.40 | 9.70 | 49.10 | 49.10 | 17.80 | 12.80 | 19.80 | 28.40 |

**Table S3.** The percentages of cells of KKU-M213B and KKU-100 were indicated apoptosis with Annexin V-FITC and Propidium iodide (PI) and analyzed by flow cytometry in duplicate.

| **Cells** | **Sample (µg/ml)** | **Exp. 1** | | **Exp.2** | |
| --- | --- | --- | --- | --- | --- |
|  |  | **Early rate** | **Late rate** | **Early rate** | **Late rate** |
| **KKU-M213B** | **Control** | 5.00 | 0 | 6.00 | 0 |
|  | **Camptothecin** | 16.00 | 0 | 17.20 | 0 |
|  | **31.25** | 10.40 | 0 | 13.70 | 0 |
|  | **62.5** | 13.30 | 0 | 17.80 | 0 |
|  | **125** | 23.60 | 0 | 27.90 | 0 |
| **KKU-100** | **Control** | 3.90 | 0.00 | 7.50 | 0 |
|  | **Camptothecin** | 37.40 | 0.00 | 37.80 | 0 |
|  | **31.25** | 7.20 | 1.20 | 13.70 | 0 |
|  | **62.5** | 8.30 | 0.10 | 15.10 | 0 |
|  | **125** | 18.90 | 0.40 | 16.60 | 0 |
| **H69** | **Control** | 4.10 | 0.00 | 3.00 | 0 |
|  | **Camptothecin** | 17.20 | 0.00 | 18.90 | 0 |
|  | **31.25** | 5.70 | 0.10 | 4.10 | 0 |
|  | **62.5** | 7.40 | 0.00 | 5.30 | 0 |
|  | **125** | 7.90 | 0.00 | 6.60 | 0 |

**Table S4.** The intensity values showed expression of p53, p21, CDK4, Bax, Ac-H3, Bcl2 and p-ERK were detected by immunoblotting in duplicate.

| **Sample (µg/ml)** | **KKU-M213B cells** | | | |
| --- | --- | --- | --- | --- |
|  | **Exp.1** | | **Exp.2** | |
|  | **Ac-H3/ERK** | **Relative fold** | **Ac-H3/ERK** | **Relative fold** |
| **control** | 0.736 | 1.000 | 0.788 | 1.000 |
| **31.25** | 0.744 | 1.011 | 0.644 | 0.818 |
| **62.50** | 0.821 | 1.116 | 0.960 | 1.219 |
| **125** | 1.160 | 1.577 | 1.316 | 1.670 |
|  | **p21/ERK** |  | **p21/ERK** |  |
| **Control** | 0.00 | 1.000 | 0.00 | 1.000 |
| **31.25** | 0.00 | 1.000 | 0.00 | 1.000 |
| **62.50** | 0.00 | 1.000 | 0.00 | 1.000 |
| **125** | 0.00 | 1.000 | 0.00 | 1.000 |
|  | **CDK4/ERK** |  | **CDK4/ERK** |  |
| **Control** | 0.92 | 1.000 | 0.92 | 1.000 |
| **31.25** | 0.72 | 0.788 | 0.75 | 0.820 |
| **62.50** | 0.68 | 0.738 | 0.70 | 0.757 |
| **125** | 0.66 | 0.719 | 0.66 | 0.721 |
|  | **p53/ERK** |  | **p53/ERK** |  |
| **Control** | 1.58 | 1.000 | 1.10 | 1.000 |
| **31.25** | 1.49 | 0.942 | 0.91 | 0.829 |
| **62.50** | 0.69 | 0.436 | 0.68 | 0.619 |
| **125** | 0.18 | 0.113 | 0.44 | 0.403 |
|  | **Bax/ERK** |  | **Bax/ERK** |  |
| **Control** | 1.13 | 1.000 | 0.74 | 1.000 |
| **31.25** | 0.79 | 0.697 | 0.61 | 0.818 |
| **62.50** | 0.46 | 0.368 | 0.64 | 0.856 |
| **125** | 0.51 | 0.453 | 0.57 | 0.769 |
|  | **Bcl2/ERK** |  | **Bcl2/ERK** |  |
| **Control** | 0.92 | 1.000 | 1.10 | 1.000 |
| **31.25** | 0.71 | 0.771 | 1.16 | 1.068 |
| **62.50** | 0.25 | 0.270 | 0.93 | 0.852 |
| **125** | 0.079 | 0.086 | 0.11 | 0.099 |
|  | **p-ERK/ERK** |  | **p-ERK/ERK** |  |
| **Control** | 1.08 | 1.000 | 1.04 | 1.000 |
| **31.25** | 1.04 | 0.965 | 1.02 | 0.972 |
| **62.50** | 0.72 | 0.672 | 0.62 | 0.596 |
| **125** | 0.64 | 0.596 | 0.47 | 0.452 |

| **Sample (µg/ml)** | **KKU-100 cells** | | | |
| --- | --- | --- | --- | --- |
|  | **Exp.1** | | **Exp.2** | |
|  | **Ac-H3/ERK** | **Relative fold** | **Ac-H3/ERK** | **Relative fold** |
| **control** | 0.154 | 1 | 0.213 | 1 |
| **31.25** | 0.174 | 1.128 | 0.210 | 0.983 |
| **62.50** | 0.400 | 2.589 | 0.519 | 2.432 |
| **125** | 1.566 | 10.148 | 1.809 | 8.485 |
|  | **p21/ERK** |  | **p21/ERK** |  |
| **Control** | 0.117 | 1.000 | 0.139 | 1.000 |
| **31.25** | 0.229 | 1.955 | 0.283 | 2.043 |
| **62.50** | 0.360 | 3.069 | 0.428 | 3.093 |
| **125** | 1.111 | 9.472 | 1.136 | 8.199 |
|  | **CDK4/ERK** |  | **CDK4/ERK** |  |
| **Control** | 1.135 | 1.000 | 1.239 | 1.000 |
| **31.25** | 0.929 | 0.819 | 0.947 | 0.764 |
| **62.50** | 0.699 | 0.616 | 0.533 | 0.430 |
| **125** | 0.454 | 0.400 | 0.758 | 0.611 |
|  | **p53/ERK** |  | **p53/ERK** |  |
| **Control** | 0.94 | 1.000 | 0.832 | 1.000 |
| **31.25** | 0.62 | 0.685 | 0.724 | 0.869 |
| **62.50** | 0.50 | 0.410 | 0.579 | 0.696 |
| **125** | 0.32 | 0.208 | 0.614 | 0.738 |
|  | **Bax/ERK** |  | **Bax/ERK** |  |
| **Control** | 0.503 | 1.000 | 0.261 | 1.000 |
| **31.25** | 0.541 | 1.077 | 0.382 | 1.464 |
| **62.50** | 0.809 | 1.609 | 0.399 | 1.533 |
| **125** | 1.480 | 2.945 | 0.696 | 2.673 |
|  | **Bcl2/ERK** |  | **Bcl2/ERK** |  |
| **Control** | 0.600 | 1.000 | 0.920 | 1.000 |
| **31.25** | 0.791 | 1.319 | 0.905 | 0.983 |
| **62.50** | 0.304 | 0.507 | 0.728 | 0.791 |
| **125** | 0.294 | 0.490 | 0.612 | 0.665 |
|  | **p-ERK/ERK** |  | **p-ERK/ERK** |  |
| **Control** | 1.153 | 1.000 | 1.232 | 1.000 |
| **31.25** | 1.134 | 0.983 | 1.200 | 0.974 |
| **62.50** | 0.837 | 0.726 | 0.960 | 0.780 |
| **125** | 0.341 | 0.296 | 0.494 | 0.401 |

**Table S5.** The intensity values showed expression of Bax/Bcl2 ratio.

| **Sample (µg/ml)** | **KKU-M213B cells** | | | | | | | |
| --- | --- | --- | --- | --- | --- | --- | --- | --- |
|  | **Exp.1** | | | | **Exp.2** | | | |
|  | **Bax/ERK** | **Bcl2/ERK** | **Bax/Bcl2** | **Relative**  **fold** | **Bax/ERK** | **Bcl2/ERK** | **Bax/Bcl2** | **Relative**  **fold** |
| **Control** | 1.129 | 0.915 | 1.234 | 1.000 | 0.742 | 1.090 | 0.681 | 1.000 |
| **31.25** | 0.787 | 0.706 | 1.115 | 0.904 | 0.607 | 1.164 | 0.521 | 0.765 |
| **62.50** | 0.416 | 0.247 | 1.682 | 1.363 | 0.635 | 0.928 | 0.684 | 1.005 |
| **125** | 0.511 | 0.079 | 6.474 | 5.246 | 0.571 | 0.108 | 5.284 | 7.759 |

| **Sample (µg/ml)** | **KKU-100 cells** | | | | | | | |
| --- | --- | --- | --- | --- | --- | --- | --- | --- |
|  | **Exp.1** | | | | **Exp.2** | | | |
|  | **Bax/ERK** | **Bcl2/ERK** | **Bax/Bcl2** | **Relative fold** | **Bax/ERK** | **Bcl2/ERK** | **Bax/Bcl2** | **Relative fold** |
| **Control** | 0.503 | 0.600 | 0.838 | 1.000 | 0.261 | 0.920 | 0.283 | 1.000 |
| **31.25** | 0.541 | 0.791 | 0.685 | 0.817 | 0.382 | 0.905 | 0.422 | 1.490 |
| **62.50** | 0.809 | 0.304 | 2.662 | 3.175 | 0.399 | 0.728 | 0.549 | 1.938 |
| **125** | 1.480 | 0.294 | 5.041 | 6.012 | 0.696 | 0.612 | 1.138 | 4.018 |
