# Supplementary material for: Ethanolic extract of Ya-nang (Tiliacora triandra) leaf powder induces apoptosis in cholangiocarcinoma cell lines via induction of hyperacetylation and inhibition of growth signaling
Source: PeerJ. 2022 Dec 15;10:e14518. doi: 10.7717/peerj.14518 (PMC9760018; doi:10.7717/peerj.14518)
Supplement: Supplemental Information 2 — Microscopic pictures of KKU-M213B and KKU-100 cells after treatments with varied concentrations of TLPE extract for 24 h. [file peerj-10-14518-s002.pdf]

KKU-M213B cells

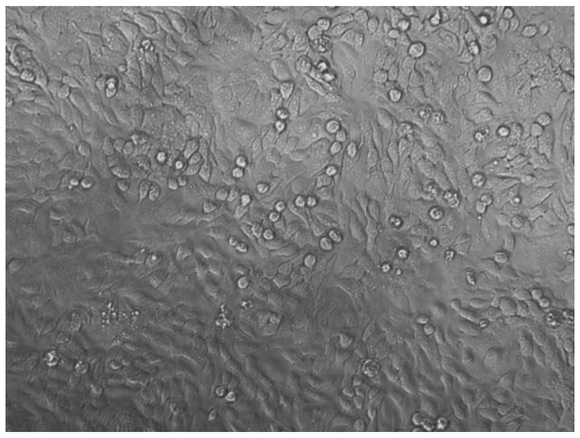

Solvent control

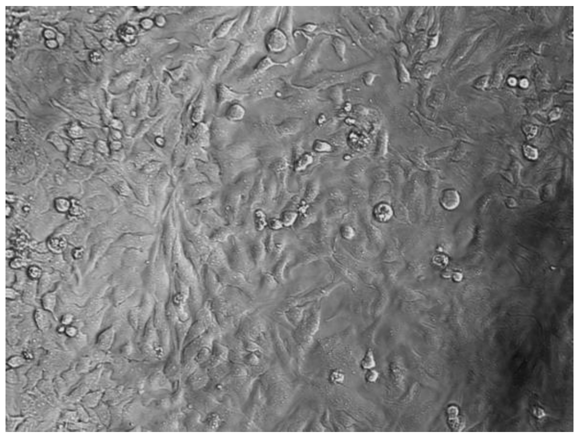

TLPE extract 31.25 μg/mL

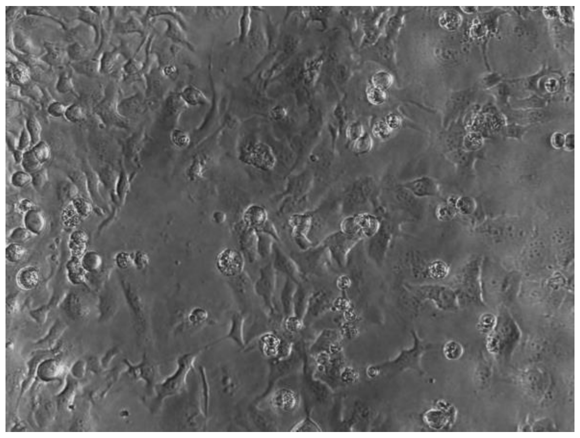

TLPE extract 62.50 μg/mL

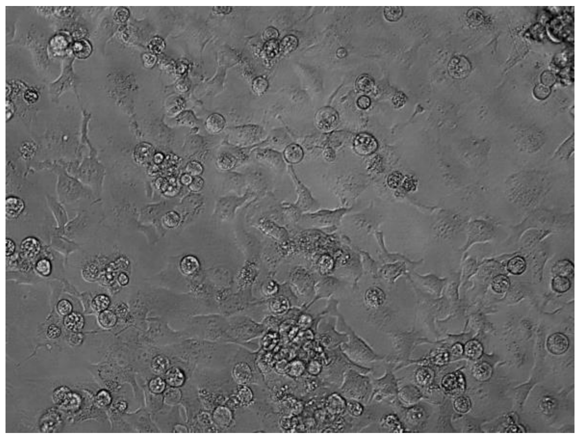

TLPE extract 125 μg/mL

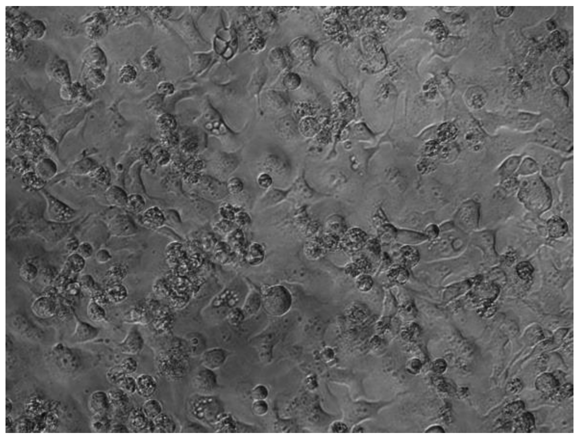

TLPE extract 250 μg/mL

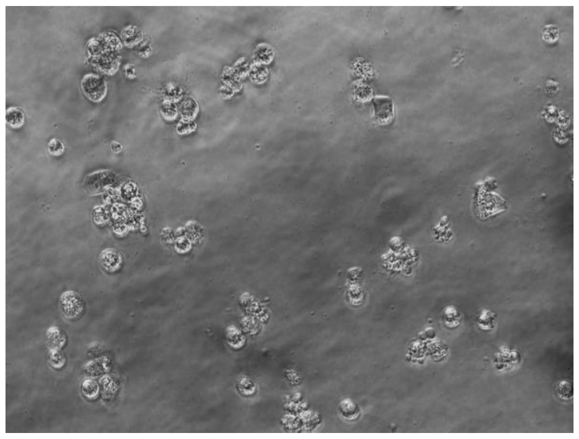

TLPE extract 500 μg/mL

KKU-100 cells

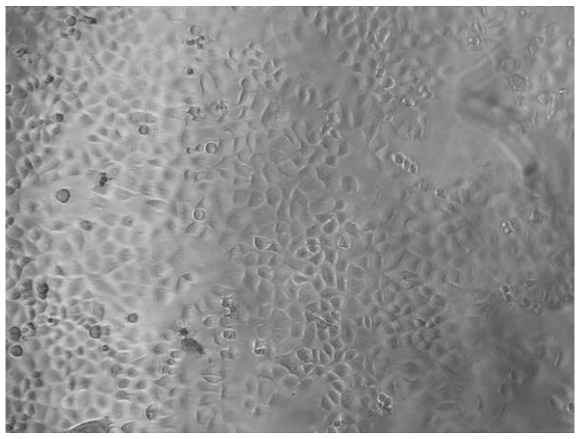

Solvent control

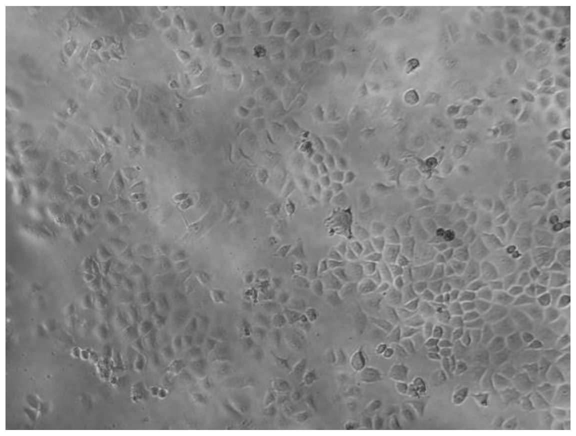

TLPE extract 31.25 μg/mL

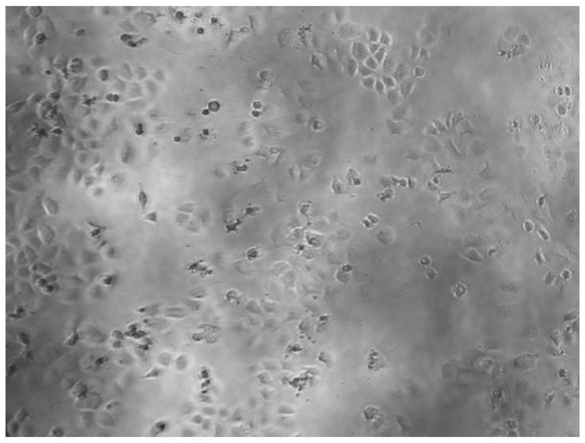

TLPE extract 62.50 μg/mL

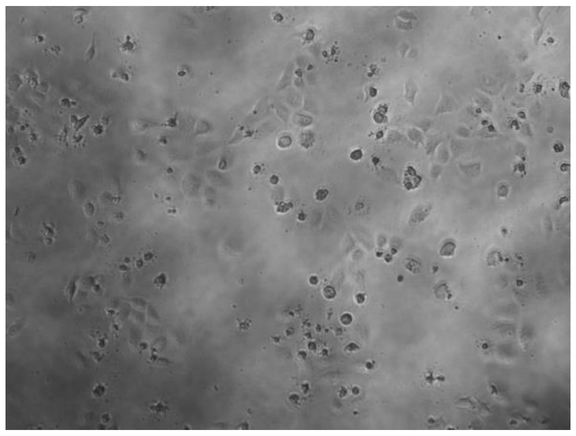

TLPE extract 125 μg/mL

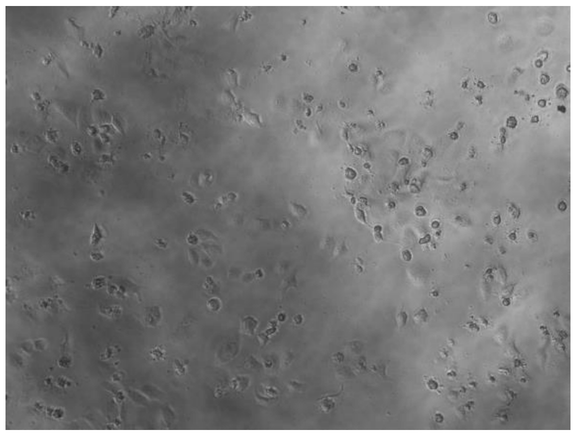

TLPE extract 250 μg/mL

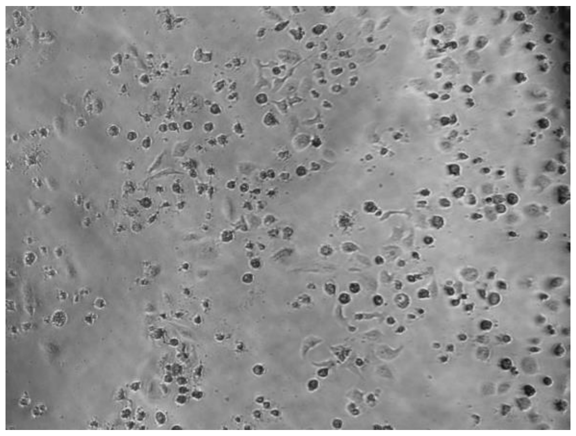

TLPE extract 500 μg/mL
