# Supplementary material for: Ethanolic extract of Ya-nang (Tiliacora triandra) leaf powder induces apoptosis in cholangiocarcinoma cell lines via induction of hyperacetylation and inhibition of growth signaling
Source: PeerJ. 2022 Dec 15;10:e14518. doi: 10.7717/peerj.14518 (PMC9760018; doi:10.7717/peerj.14518)
Supplement: Supplemental Information 3 [file peerj-10-14518-s003.pdf]

1. **SPSS data of cell viability on KKU-M213B, KKU-100, and H69 cell lines.**

**1.1 KKU-M213B cells**

**1.1.1 24 h**

**ANOVA**

**KKU-M213B cells**

| | Sum of Squares | df | Mean Square | F | Sig. |
|---|---|---|---|---|---|
| Between Groups | 11603.526 | 5 | 2320.705 | 65.263 | .000 |
| Within Groups | 213.354 | 6 | 35.559 | | |
| Total | 11816.880 | 11 | | | |

Duncan[a]

| TLPE extract | N | Subset for alpha = 0.05 | | | | |
|---|---|---|---|---|---|---|
| | | 1 | 2 | 3 | 4 | 5 |
| 500 | 2 | 8.5725 | | | | |
| 250 | 2 | | 30.8670 | | | |
| 125 | 2 | | 34.3990 | | | |
| 62.5 | 2 | | | 62.3005 | | |
| 31.25 | 2 | | | | 79.4120 | |
| solvent control | 2 | | | | | 100.0000 |
| Sig. | | 1.000 | .575 | 1.000 | 1.000 | 1.000 |

Means for groups in homogeneous subsets are displayed.

a. Uses Harmonic Mean Sample Size = 2.000.

**1.1.2 48 h**

**ANOVA**

**KKU-M213B cells**

| | Sum of Squares | df | Mean Square | F | Sig. |
|---|---|---|---|---|---|
| Between Groups | 26265.908 | 7 | 3752.273 | 197.363 | .000 |
| Within Groups | 152.097 | 8 | 19.012 | | |
| Total | 26418.005 | 15 | | | |

Duncan[a]

| TLPE extract | N | Subset for alpha = 0.05 | | | |
|---|---|---|---|---|---|
| | | 1 | 2 | 3 | 4 |
| 12 | 2 | 1.1765 | | | |
| 15 | 2 | 1.3965 | | | |
| 30 | 2 | 1.4410 | | | |
| 10 | 2 | 1.9310 | | | |
| 9 | 2 | | 55.4655 | | |
| 7.5 | 2 | | | 67.7045 | |
| 3.75 | 2 | | | | 90.9185 |
| solvent control | 2 | | | | 100.0000 |
| Sig. | | .874 | 1.000 | 1.000 | .071 |

Means for groups in homogeneous subsets are displayed.

a. Uses Harmonic Mean Sample Size = 2.000.

**1.1.3 72 h**

**ANOVA**

**KKU-M213B cells**

| | Sum of Squares | df | Mean Square | F | Sig. |
|---|---|---|---|---|---|
| Between Groups | 24950.598 | 7 | 3564.371 | 161.449 | .000 |
| Within Groups | 176.619 | 8 | 22.077 | | |
| Total | 25127.216 | 15 | | | |

Duncan[a]

| | | Subset for alpha = 0.05 | | | | |
|---|---|---|---|---|---|---|
| TLPE extract | N | 1 | 2 | 3 | 4 | 5 |
| 12 | 2 | .8395 | | | | |
| 10 | 2 | 1.0505 | | | | |
| 30 | 2 | 1.1230 | | | | |
| 15 | 2 | 1.3100 | | | | |
| 9 | 2 | | 14.8365 | | | |
| 7.5 | 2 | | | 60.9085 | | |
| 3.75 | 2 | | | | 85.9390 | |
| solvent control | 2 | | | | | 100.0000 |
| Sig. | | .927 | 1.000 | 1.000 | 1.000 | 1.000 |

Means for groups in homogeneous subsets are displayed.

a. Uses Harmonic Mean Sample Size = 2.000.

## 1.2 KKU-100 cells

### 1.2.1 24 h

**ANOVA**

**KKU-100 cells**

| | Sum of Squares | df | Mean Square | F | Sig. |
|---|---|---|---|---|---|
| Between Groups | 11818.836 | 5 | 2363.767 | 851.584 | .000 |
| Within Groups | 16.654 | 6 | 2.776 | | |
| Total | 11835.490 | 11 | | | |

Duncan[a]

| | | Subset for alpha = 0.05 | | | | |
|---|---|---|---|---|---|---|
| TLPE extract | N | 1 | 2 | 3 | 4 | 5 |
| 500 | 2 | 3.7240 | | | | |
| 250 | 2 | | 45.3245 | | | |
| 125 | 2 | | 48.0310 | | | |
| 62.5 | 2 | | | 59.1465 | | |
| 31.25 | 2 | | | | 88.6560 | |
| solvent control | 2 | | | | | 100.0000 |
| Sig. | | 1.000 | .155 | 1.000 | 1.000 | 1.000 |

Means for groups in homogeneous subsets are displayed.

a. Uses Harmonic Mean Sample Size = 2.000.

**1.2.2 48 h**

**ANOVA**

**KKU-100 cells**

| | Sum of Squares | df | Mean Square | F | Sig. |
|---|---|---|---|---|---|
| Between Groups | 14701.493 | 7 | 2100.213 | 77.785 | .000 |
| Within Groups | 216.002 | 8 | 27.000 | | |
| Total | 14917.495 | 15 | | | |

Duncan[a]

| | | Subset for alpha = 0.05 | | | |
|---|---|---|---|---|---|
| TLPE extract | N | 1 | 2 | 3 | 4 |
| 30 | 2 | 10.1025 | | | |
| 15 | 2 | | 40.3710 | | |
| 12 | 2 | | | 61.5495 | |
| 7.5 | 2 | | | | 91.1220 |
| 9 | 2 | | | | 91.4395 |
| 10 | 2 | | | | 91.5375 |
| 3.75 | 2 | | | | 94.4315 |
| solvent control | 2 | | | | 100.0000 |
| Sig. | | 1.000 | 1.000 | 1.000 | .150 |

Means for groups in homogeneous subsets are displayed.

a. Uses Harmonic Mean Sample Size = 2.000.

**1.2.3 72 h**

**ANOVA**

**KKU-100 cells**

| | Sum of Squares | df | Mean Square | F | Sig. |
|---|---|---|---|---|---|
| Between Groups | 25156.769 | 7 | 3593.824 | 606.081 | .000 |
| Within Groups | 47.437 | 8 | 5.930 | | |
| Total | 25204.206 | 15 | | | |

Duncan[a]

| | | Subset for alpha = 0.05 | | | | |
|---|---|---|---|---|---|---|
| TLPE extract | N | 1 | 2 | 3 | 4 | 5 |
| 30 | 2 | 1.6850 | | | | |
| 15 | 2 | 3.7245 | | | | |
| 12 | 2 | 5.8330 | | | | |
| 10 | 2 | | 24.3595 | | | |
| 9 | 2 | | | 43.3820 | | |
| 7.5 | 2 | | | | 67.0135 | |
| solvent control | 2 | | | | | 100.0000 |
| 3.75 | 2 | | | | | 104.4775 |
| Sig. | | .141 | 1.000 | 1.000 | 1.000 | .103 |

Means for groups in homogeneous subsets are displayed.

a. Uses Harmonic Mean Sample Size = 2.000.

## 1.3 H69 cells

### 1.3.1 24 h

**ANOVA**

**H69 cells**

| | Sum of Squares | df | Mean Square | F | Sig. |
|---|---|---|---|---|---|
| Between Groups | 11013.466 | 5 | 2202.693 | 224.285 | .000 |
| Within Groups | 58.926 | 6 | 9.821 | | |
| Total | 11072.391 | 11 | | | |

Duncan[a]

| | | Subset for alpha = 0.05 | | | | | |
|---|---|---|---|---|---|---|---|
| TLPE extract | N | 1 | 2 | 3 | 4 | 5 | 6 |
| 500 | 2 | 16.8755 | | | | | |
| 250 | 2 | | 30.5060 | | | | |
| 125 | 2 | | | 53.2495 | | | |
| 62.5 | 2 | | | | 77.7240 | | |
| 31.25 | 2 | | | | | 88.7365 | |
| solvent control | 2 | | | | | | 100.0000 |
| Sig. | | 1.000 | 1.000 | 1.000 | 1.000 | 1.000 | 1.000 |

Means for groups in homogeneous subsets are displayed.

a. Uses Harmonic Mean Sample Size = 2.000.

**1.3.2 48 h**

**ANOVA**

**H69 cells**

| | Sum of Squares | df | Mean Square | F | Sig. |
|---|---|---|---|---|---|
| Between Groups | 6515.345 | 7 | 930.764 | 86.997 | .000 |
| Within Groups | 85.590 | 8 | 10.699 | | |
| Total | 6600.936 | 15 | | | |

Duncan[a]

| | | Subset for alpha = 0.05 | | | | | |
|---|---|---|---|---|---|---|---|
| TLPE extract | N | 1 | 2 | 3 | 4 | 5 | 6 |
| 30 | 2 | 22.5375 | | | | | |
| 15 | 2 | | 48.6780 | | | | |
| 12 | 2 | | 54.9245 | 54.9245 | | | |
| 10 | 2 | | 56.0240 | 56.0240 | | | |
| 9 | 2 | | | 60.2800 | 60.2800 | | |
| 7.5 | 2 | | | | 63.9675 | 63.9675 | |
| 3.75 | 2 | | | | | 68.4805 | |
| solvent control | 2 | | | | | | 100.0000 |
| Sig. | | 1.000 | .063 | .155 | .292 | .205 | 1.000 |

Means for groups in homogeneous subsets are displayed.

a. Uses Harmonic Mean Sample Size = 2.000.

**1.3.3 72 h**

**ANOVA**

**H69 cells**

| | Sum of Squares | df | Mean Square | F | Sig. |
|---|---|---|---|---|---|
| Between Groups | 9573.510 | 7 | 1367.644 | 37.695 | .000 |
| Within Groups | 290.256 | 8 | 36.282 | | |
| Total | 9863.766 | 15 | | | |

Duncan[a]

| | | Subset for alpha = 0.05 | | | | |
|---|---|---|---|---|---|---|
| TLPE extract | N | 1 | 2 | 3 | 4 | 5 |
| 30 | 2 | 12.2630 | | | | |
| 15 | 2 | | 38.9850 | | | |
| 12 | 2 | | 49.5545 | 49.5545 | | |
| 10 | 2 | | | 62.6040 | 62.6040 | |
| 9 | 2 | | | 63.1185 | 63.1185 | |
| 7.5 | 2 | | | | 68.3125 | |
| 3.75 | 2 | | | | 76.7465 | |
| solvent control | 2 | | | | | 100.0000 |
| Sig. | | 1.000 | .117 | .063 | .059 | 1.000 |

Means for groups in homogeneous subsets are displayed.

a. Uses Harmonic Mean Sample Size = 2.000.

2. **SPSS data of cell cycle arrest analysis on KKU-M213B, KKU-100, and H69 cell lines.**

## 2.1 KKU-M213B cells

### 2.1.1 SubG1 phase

**ANOVA**

**KKU-M213B cells**

| | Sum of Squares | df | Mean Square | F | Sig. |
|---|---|---|---|---|---|
| Between Groups | 197.994 | 3 | 65.998 | 28.401 | .004 |
| Within Groups | 9.295 | 4 | 2.324 | | |
| Total | 207.289 | 7 | | | |

Duncan[a]

| | | Subset for alpha = 0.05 | | |
|---|---|---|---|---|
| TLPE extract | N | 1 | 2 | 3 |
| solvent control | 2 | 6.6000 | | |
| 31.25 | 2 | | 12.2500 | |
| 62.5 | 2 | | | 17.0500 |
| 125 | 2 | | | 19.6500 |
| Sig. | | 1.000 | 1.000 | .163 |

Means for groups in homogeneous subsets are displayed.

a. Uses Harmonic Mean Sample Size = 2.000.

### 2.1.2 G0/G1 phase

**ANOVA**

**KKU-M213B cells**

| | Sum of Squares | df | Mean Square | F | Sig. |
|---|---|---|---|---|---|
| Between Groups | 147.724 | 3 | 49.241 | 37.128 | .002 |
| Within Groups | 5.305 | 4 | 1.326 | | |
| Total | 153.029 | 7 | | | |

Duncan[a]

| TLPE extract | N | Subset for alpha = 0.05 | |
|---|---|---|---|
| | | 1 | 2 |
| 125 | 2 | 67.9000 | |
| 62.5 | 2 | | 77.2500 |
| 31.25 | 2 | | 77.4000 |
| solvent control | 2 | | 78.6000 |
| Sig. | | 1.000 | .312 |

Means for groups in homogeneous subsets are displayed.

a. Uses Harmonic Mean Sample Size = 2.000.

### 2.1.3 S phase

**ANOVA**

**KKU-M213B cells**

| | Sum of Squares | df | Mean Square | F | Sig. |
|---|---|---|---|---|---|
| Between Groups | 6.664 | 3 | 2.221 | 6.415 | .052 |
| Within Groups | 1.385 | 4 | .346 | | |
| Total | 8.049 | 7 | | | |

Duncan[a]

| TLPE extract | N | Subset for alpha = 0.05 1 | 2 |
|---|---|---|---|
| 62.5 | 2 | 2.9000 | |
| 31.25 | 2 | 4.4000 | 4.4000 |
| solvent control | 2 | 4.5000 | 4.5000 |
| 125 | 2 | | 5.4500 |
| Sig. | | .056 | .154 |

Means for groups in homogeneous subsets are displayed.
a. Uses Harmonic Mean Sample Size = 2.000.

### 2.1.4 G2/M phase

**ANOVA**

**KKU-M213B cells**

| | Sum of Squares | df | Mean Square | F | Sig. |
|---|---|---|---|---|---|
| Between Groups | 58.650 | 3 | 19.550 | 9.082 | .029 |
| Within Groups | 8.610 | 4 | 2.153 | | |
| Total | 67.260 | 7 | | | |

Duncan[a]

| TLPE extract | N | Subset for alpha = 0.05 1 | 2 |
|---|---|---|---|
| 62.5 | 2 | 2.7000 | |
| 31.25 | 2 | 5.8500 | |
| 125 | 2 | 6.7500 | 6.7500 |
| solvent control | 2 | | 10.3000 |
| Sig. | | .054 | .073 |

Means for groups in homogeneous subsets are displayed.
a. Uses Harmonic Mean Sample Size = 2.000.

## 2.2 KKU-100 cells

### 2.2.1 SubG1 phase

**ANOVA**

**KKU-100 cells**

| | Sum of Squares | df | Mean Square | F | Sig. |
|---|---|---|---|---|---|
| Between Groups | 55.634 | 3 | 18.545 | 449.566 | .000 |
| Within Groups | .165 | 4 | .041 | | |
| Total | 55.799 | 7 | | | |

Duncan[a]

| | | Subset for alpha = 0.05 | | |
|---|---|---|---|---|
| TLPE extract | N | 1 | 2 | 3 |
| solvent control | 2 | .8000 | | |
| 31.25 | 2 | 1.3000 | 1.3000 | |
| 62.5 | 2 | | 1.6500 | |
| 125 | 2 | | | 7.3000 |
| Sig. | | .070 | .160 | 1.000 |

Means for groups in homogeneous subsets are displayed.

a. Uses Harmonic Mean Sample Size = 2.000.

**2.2.2 G0/G1 phase**

**ANOVA**

**KKU-100 cells**

| | Sum of Squares | df | Mean Square | F | Sig. |
|---|---|---|---|---|---|
| Between Groups | 202.690 | 3 | 67.563 | 34.166 | .003 |
| Within Groups | 7.910 | 4 | 1.977 | | |
| Total | 210.600 | 7 | | | |

Duncan[a]

| TLPE extract | N | Subset for alpha = 0.05 | |
|---|---|---|---|
| | | 1 | 2 |
| solvent control | 2 | 72.0000 | |
| 31.25 | 2 | | 82.9000 |
| 62.5 | 2 | | 83.1500 |
| 125 | 2 | | 84.5500 |
| Sig. | | 1.000 | .312 |

Means for groups in homogeneous subsets are displayed.
a. Uses Harmonic Mean Sample Size = 2.000.

**2.2.3 S phase**

**ANOVA**

**KKU-100 cells**

| | Sum of Squares | df | Mean Square | F | Sig. |
|---|---|---|---|---|---|
| Between Groups | 72.024 | 3 | 24.008 | 35.241 | .002 |
| Within Groups | 2.725 | 4 | .681 | | |
| Total | 74.749 | 7 | | | |

Duncan[a]

| | | Subset for alpha = 0.05 | | |
|---|---|---|---|---|
| TLPE extract | N | 1 | 2 | 3 |
| 125 | 2 | 3.9500 | | |
| 62.5 | 2 | | 6.4000 | |
| 31.25 | 2 | | 7.9000 | |
| solvent control | 2 | | | 12.2000 |
| Sig. | | 1.000 | .143 | 1.000 |

Means for groups in homogeneous subsets are displayed.

a. Uses Harmonic Mean Sample Size = 2.000.

**2.2.4 G2/M phase**

**ANOVA**

**KKU-100 cells**

| | Sum of Squares | df | Mean Square | F | Sig. |
|---|---|---|---|---|---|
| Between Groups | 110.704 | 3 | 36.901 | 13.316 | .015 |
| Within Groups | 11.085 | 4 | 2.771 | | |
| Total | 121.789 | 7 | | | |

Duncan[a]

| TLPE extract | N | Subset for alpha = 0.05 1 | 2 |
|---|---|---|---|
| 125 | 2 | 4.2000 | |
| 62.5 | 2 | 7.4500 | |
| 31.25 | 2 | 7.5000 | |
| solvent control | 2 | | 14.4000 |
| Sig. | | .124 | 1.000 |

Means for groups in homogeneous subsets are displayed.
a. Uses Harmonic Mean Sample Size = 2.000.

## 2.3 H69 cells

### 2.3.1 SubG1

**ANOVA**

**H69 cells**

| | Sum of Squares | df | Mean Square | F | Sig. |
|---|---|---|---|---|---|
| Between Groups | 38.925 | 3 | 12.975 | 21.535 | .006 |
| Within Groups | 2.410 | 4 | .603 | | |
| Total | 41.335 | 7 | | | |

Duncan[a]

| TLPE | N | Subset for alpha = 0.05 | |
|---|---|---|---|
| | | 1 | 2 |
| solvent control | 2 | 3.7000 | |
| 31.25 | 2 | | 7.3500 |
| 62.5 | 2 | | 8.5000 |
| 125 | 2 | | 9.5500 |
| Sig. | | 1.000 | .050 |

Means for groups in homogeneous subsets are displayed.
a. Uses Harmonic Mean Sample Size = 2.000.

**2.3.2 G0/G1 phase**

**ANOVA**

**H69 cells**

| | Sum of Squares | df | Mean Square | F | Sig. |
|---|---|---|---|---|---|
| Between Groups | 28.084 | 3 | 9.361 | 1.407 | .363 |
| Within Groups | 26.605 | 4 | 6.651 | | |
| Total | 54.689 | 7 | | | |

Duncan[a]

| | | Subset for alpha = 0.05 |
|---|---|---|
| TLPE extract | N | 1 |
| solvent control | 2 | 46.2500 |
| 31.25 | 2 | 48.4000 |
| 125 | 2 | 49.1000 |
| 62.5 | 2 | 51.5000 |
| Sig. | | .117 |

Means for groups in homogeneous subsets are displayed.

a. Uses Harmonic Mean Sample Size = 2.000.

### 2.3.3 S phase

**ANOVA**

**H69 cells**

| | Sum of Squares | df | Mean Square | F | Sig. |
|---|---|---|---|---|---|
| Between Groups | .664 | 3 | .221 | .009 | .999 |
| Within Groups | 103.645 | 4 | 25.911 | | |
| Total | 104.309 | 7 | | | |

Duncan[a]

| TLPE extract | N | Subset for alpha = 0.05<br>1 |
|---|---|---|
| 62.5 | 2 | 14.5000 |
| solvent control | 2 | 14.8500 |
| 31.25 | 2 | 15.0000 |
| 125 | 2 | 15.3000 |
| Sig. | | .883 |

Means for groups in homogeneous subsets are displayed.
a. Uses Harmonic Mean Sample Size = 2.000.

**2.3.4 G2/M phase**

**ANOVA**

**H69 cells**

| | Sum of Squares | df | Mean Square | F | Sig. |
|---|---|---|---|---|---|
| Between Groups | 79.495 | 3 | 26.498 | .579 | .659 |
| Within Groups | 183.000 | 4 | 45.750 | | |
| Total | 262.495 | 7 | | | |

Duncan[a]

| TLPE extract | N | Subset for alpha = 0.05<br>1 |
|---|---|---|
| 62.5 | 2 | 23.7000 |
| 125 | 2 | 24.1000 |
| 31.25 | 2 | 26.9000 |
| solvent control | 2 | 31.6000 |
| Sig. | | .312 |

Means for groups in homogeneous subsets are displayed.

a. Uses Harmonic Mean Sample Size = 2.000.

**3. SPSS data of apoptosis analysis on KKU-M213B, KKU-100, and H69 cell lines.**

**3.1 KKU-M213B cells**

**ANOVA**

**KKU-M213B cells**

| | Sum of Squares | df | Mean Square | F | Sig. |
|---|---|---|---|---|---|
| Between Groups | 434.674 | 4 | 108.669 | 20.870 | .003 |
| Within Groups | 26.035 | 5 | 5.207 | | |
| Total | 460.709 | 9 | | | |

Duncan[a]

| | | Subset for alpha = 0.05 | | |
|---|---|---|---|---|
| TLPE extract | N | 1 | 2 | 3 |
| solvent control | 2 | 5.5000 | | |
| 31.25 | 2 | | 12.0500 | |
| 62.5 | 2 | | 15.5500 | |
| camp | 2 | | 16.6000 | |
| 125 | 2 | | | 25.7500 |
| Sig. | | 1.000 | .110 | 1.000 |

Means for groups in homogeneous subsets are displayed.

a. Uses Harmonic Mean Sample Size = 2.000.

**3.2 KKU-100 cells**

**ANOVA**

**KKU-100 cells**

| | Sum of Squares | df | Mean Square | F | Sig. |
|---|---|---|---|---|---|
| Between Groups | 1245.914 | 4 | 311.478 | 29.137 | .001 |
| Within Groups | 53.450 | 5 | 10.690 | | |
| Total | 1299.364 | 9 | | | |

Duncan[a]

| | | Subset for alpha = 0.05 | | |
|---|---|---|---|---|
| TLPE extract | N | 1 | 2 | 3 |
| solvent control | 2 | 5.7000 | | |
| 31.25 | 2 | 10.4500 | 10.4500 | |
| 62.5 | 2 | 11.7000 | 11.7000 | |
| 125 | 2 | | 17.7500 | |
| camp | 2 | | | 37.6000 |
| Sig. | | .134 | .082 | 1.000 |

Means for groups in homogeneous subsets are displayed.

a. Uses Harmonic Mean Sample Size = 2.000.

**3.3 H69 cells**

**ANOVA**

**H69 cells**

| | Sum of Squares | df | Mean Square | F | Sig. |
|---|---|---|---|---|---|
| Between Groups | 267.396 | 4 | 66.849 | 52.389 | .000 |
| Within Groups | 6.380 | 5 | 1.276 | | |
| Total | 273.776 | 9 | | | |

Duncan[a]

| TLPE extract | N | Subset for alpha = 0.05 1 | 2 | 3 |
|---|---|---|---|---|
| solvent control | 2 | 3.5500 | | |
| 31.25 | 2 | 4.9000 | 4.9000 | |
| 62.5 | 2 | 6.3500 | 6.3500 | |
| 125 | 2 | | 7.2500 | |
| camp | 2 | | | 18.0500 |
| Sig. | | .061 | .099 | 1.000 |

Means for groups in homogeneous subsets are displayed.

a. Uses Harmonic Mean Sample Size = 2.000.

## 4. SPSS data of western blot analysis on KKU-M213B and KKU-100 cell lines.

### 4.1 KKU-M213B cells

#### 4.1.1 Ac-H3

**ANOVA**

**KKU-M213B cells**

| | Sum of Squares | df | Mean Square | F | Sig. |
|---|---|---|---|---|---|
| Between Groups | .599 | 3 | .200 | 28.285 | .004 |
| Within Groups | .028 | 4 | .007 | | |
| Total | .628 | 7 | | | |

Duncan[a]

| | | Subset for alpha = 0.05 | | |
|---|---|---|---|---|
| TLPE extract | N | 1 | 2 | 3 |
| 31.25 | 2 | .9145 | | |
| solvent control | 2 | 1.0000 | 1.0000 | |
| 62.5 | 2 | | 1.1675 | |
| 125 | 2 | | | 1.6235 |
| Sig. | | .367 | .117 | 1.000 |

Means for groups in homogeneous subsets are displayed.

a. Uses Harmonic Mean Sample Size = 2.000.

#### 4.1.2 P21

**ANOVA**

**KKU-M213B cells**

| | Sum of Squares | df | Mean Square | F | Sig. |
|---|---|---|---|---|---|
| Between Groups | .000 | 3 | .000 | . | . |
| Within Groups | .000 | 4 | .000 | | |
| Total | .000 | 7 | | | |

### 4.1.3 CDK4

**ANOVA**

**KKU-M213B cells**

| | Sum of Squares | df | Mean Square | F | Sig. |
|---|---|---|---|---|---|
| Between Groups | .096 | 3 | .032 | 183.899 | .000 |
| Within Groups | .001 | 4 | .000 | | |
| Total | .096 | 7 | | | |

Duncan[a]

| | | Subset for alpha = 0.05 | | |
|---|---|---|---|---|
| TLPE extract | N | 1 | 2 | 3 |
| 125 | 2 | .7200 | | |
| 62.5 | 2 | .7475 | | |
| 31.25 | 2 | | .8040 | |
| solvent control | 2 | | | 1.0000 |
| Sig. | | .105 | 1.000 | 1.000 |

Means for groups in homogeneous subsets are displayed.

a. Uses Harmonic Mean Sample Size = 2.000.

**4.1.4 P53**

**ANOVA**

**KKU-M213B cells**

| | Sum of Squares | df | Mean Square | F | Sig. |
|---|---|---|---|---|---|
| Between Groups | .691 | 3 | .230 | 14.130 | .014 |
| Within Groups | .065 | 4 | .016 | | |
| Total | .756 | 7 | | | |

Duncan[a]

| TLPE extract | N | Subset for alpha = 0.05 | |
|---|---|---|---|
| | | 1 | 2 |
| 125 | 2 | .2580 | |
| 62.5 | 2 | .5275 | |
| 31.25 | 2 | | .8855 |
| solvent control | 2 | | 1.0000 |
| Sig. | | .102 | .420 |

Means for groups in homogeneous subsets are displayed.

a. Uses Harmonic Mean Sample Size = 2.000.

**4.1.5 Bax**

**ANOVA**

**KKU-M213B cells**

| | Sum of Squares | df | Mean Square | F | Sig. |
|---|---|---|---|---|---|
| Between Groups | .202 | 3 | .067 | 1.525 | .338 |
| Within Groups | .176 | 4 | .044 | | |
| Total | .378 | 7 | | | |

Duncan[a]

| factor | N | Subset for alpha = 0.05 |
|---|---|---|
| | | 1 |
| 4.00 | 2 | .6110 |
| 3.00 | 2 | .6120 |
| 2.00 | 2 | .7575 |
| 1.00 | 2 | 1.0000 |
| Sig. | | .143 |

Means for groups in homogeneous subsets are displayed.
a. Uses Harmonic Mean Sample Size = 2.000.

### 4.1.6 Bcl2

**ANOVA**

**KKU-M213B cells**

| | Sum of Squares | df | Mean Square | F | Sig. |
|---|---|---|---|---|---|
| Between Groups | 1.027 | 3 | .342 | 6.414 | .052 |
| Within Groups | .214 | 4 | .053 | | |
| Total | 1.241 | 7 | | | |

Duncan[a]

| factor | N | Subset for alpha = 0.05 | |
|---|---|---|---|
| | | 1 | 2 |
| 4.00 | 2 | .0925 | |
| 3.00 | 2 | .5610 | .5610 |
| 2.00 | 2 | | .9195 |
| 1.00 | 2 | | 1.0000 |
| Sig. | | .113 | .135 |

Means for groups in homogeneous subsets are displayed.

a. Uses Harmonic Mean Sample Size = 2.000.

### 4.1.7 pERK

**ANOVA**

**KKU-M213B cells**

| | Sum of Squares | df | Mean Square | F | Sig. |
|---|---|---|---|---|---|
| Between Groups | .358 | 3 | .119 | 48.030 | .001 |
| Within Groups | .010 | 4 | .002 | | |
| Total | .368 | 7 | | | |

Duncan[a]

| TLPE extract | N | Subset for alpha = 0.05<br>1 | 2 |
|---|---|---|---|
| 125 | 2 | .5100 | |
| 62.5 | 2 | .6330 | |
| 31.25 | 2 | | .9700 |
| solvent control | 2 | | 1.0000 |
| Sig. | | .069 | .580 |

Means for groups in homogeneous subsets are displayed.

a. Uses Harmonic Mean Sample Size = 2.000.

## 4.2 KKU-100 cells

### 4.2.1 Ac-H3

**ANOVA**

**KKU-100 cells**

| | Sum of Squares | df | Mean Square | F | Sig. |
|---|---|---|---|---|---|
| Between Groups | 94.069 | 3 | 31.356 | 89.174 | .000 |
| Within Groups | 1.407 | 4 | .352 | | |
| Total | 95.475 | 7 | | | |

Duncan[a]

| TLPE extract | N | Subset for alpha = 0.05 1 | 2 |
|---|---|---|---|
| solvent control | 2 | 1.0000 | |
| 31.25 | 2 | 1.0550 | |
| 62.5 | 2 | 2.5110 | |
| 125 | 2 | | 9.3165 |
| Sig. | | .067 | 1.000 |

Means for groups in homogeneous subsets are displayed.
a. Uses Harmonic Mean Sample Size = 2.000.

**4.2.2 P21**

**ANOVA**

**KKU-100 cells**

| | Sum of Squares | df | Mean Square | F | Sig. |
|---|---|---|---|---|---|
| Between Groups | 73.876 | 3 | 24.625 | 121.567 | .000 |
| Within Groups | .810 | 4 | .203 | | |
| Total | 74.686 | 7 | | | |

Duncan[a]

| | | Subset for alpha = 0.05 | | |
|---|---|---|---|---|
| TLPE extract | N | 1 | 2 | 3 |
| solvent control | 2 | 1.0000 | | |
| 31.25 | 2 | 1.9975 | 1.9975 | |
| 62.5 | 2 | | 3.0800 | |
| 125 | 2 | | | 8.8350 |
| Sig. | | .091 | .074 | 1.000 |

Means for groups in homogeneous subsets are displayed.

a. Uses Harmonic Mean Sample Size = 2.000.

### 4.2.3 CDK4

**ANOVA**

**KKU-100 cells**

| | Sum of Squares | df | Mean Square | F | Sig. |
|---|---|---|---|---|---|
| Between Groups | .335 | 3 | .112 | 10.871 | .022 |
| Within Groups | .041 | 4 | .010 | | |
| Total | .376 | 7 | | | |

Duncan[a]

| | | Subset for alpha = 0.05 | |
|---|---|---|---|
| TLPE extract | N | 1 | 2 |
| 125 | 2 | .5055 | |
| 62.5 | 2 | .5230 | |
| 31.25 | 2 | .7915 | .7915 |
| solvent control | 2 | | 1.0000 |
| Sig. | | .051 | .109 |

Means for groups in homogeneous subsets are displayed.

a. Uses Harmonic Mean Sample Size = 2.000.

**4.2.4 P53**

**ANOVA**

**KKU-100 cells**

| | Sum of Squares | df | Mean Square | F | Sig. |
|---|---|---|---|---|---|
| Between Groups | .337 | 3 | .112 | 2.280 | .221 |
| Within Groups | .197 | 4 | .049 | | |
| Total | .534 | 7 | | | |

Duncan[a]

| TLPE extract | N | Subset for alpha = 0.05<br>1 |
|---|---|---|
| 125 | 2 | .4740 |
| 62.5 | 2 | .5530 |
| 31.25 | 2 | .7770 |
| solvent control | 2 | 1.0000 |
| Sig. | | .082 |

Means for groups in homogeneous subsets are displayed.

a. Uses Harmonic Mean Sample Size = 2.000.

**4.2.5 Bax**

**ANOVA**

**KKU-100 cells**

| | Sum of Squares | df | Mean Square | F | Sig. |
|---|---|---|---|---|---|
| Between Groups | 3.831 | 3 | 1.277 | 44.506 | .002 |
| Within Groups | .115 | 4 | .029 | | |
| Total | 3.946 | 7 | | | |

Duncan[a]

| TLPE extract | N | Subset for alpha = 0.05 | | |
|---|---|---|---|---|
| | | 1 | 2 | 3 |
| solvent control | 2 | 1.0000 | | |
| 31.25 | 2 | 1.2705 | 1.2705 | |
| 62.5 | 2 | | 1.5710 | |
| 125 | 2 | | | 2.8090 |
| Sig. | | .186 | .151 | 1.000 |

Means for groups in homogeneous subsets are displayed.

a. Uses Harmonic Mean Sample Size = 2.000.

**4.2.6 Bcl2**

**ANOVA**

**KKU-100 cells**

| | Sum of Squares | df | Mean Square | F | Sig. |
|---|---|---|---|---|---|
| Between Groups | .458 | 3 | .153 | 5.490 | .067 |
| Within Groups | .111 | 4 | .028 | | |
| Total | .569 | 7 | | | |

Duncan[a]

| | | Subset for alpha = 0.05 | |
|---|---|---|---|
| TLPE extract | N | 1 | 2 |
| 125 | 2 | .5750 | |
| 62.5 | 2 | .6490 | |
| solvent control | 2 | 1.0000 | 1.0000 |
| 31.25 | 2 | | 1.1510 |
| Sig. | | .067 | .416 |

Means for groups in homogeneous subsets are displayed.

a. Uses Harmonic Mean Sample Size = 2.000.

### 4.2.7 pERK

**ANOVA**

**KKU-100 cells**

| | Sum of Squares | df | Mean Square | F | Sig. |
|---|---|---|---|---|---|
| Between Groups | .549 | 3 | .183 | 104.340 | .000 |
| Within Groups | .007 | 4 | .002 | | |
| Total | .556 | 7 | | | |

Duncan[a]

| TLPE extract | N | Subset for alpha = 0.05 | | |
|---|---|---|---|---|
| | | 1 | 2 | 3 |
| 125 | 2 | .3485 | | |
| 62.5 | 2 | | .7530 | |
| 31.25 | 2 | | | .9785 |
| solvent control | 2 | | | 1.0000 |
| Sig. | | 1.000 | 1.000 | .635 |

Means for groups in homogeneous subsets are displayed.
a. Uses Harmonic Mean Sample Size = 2.000.

### 4.3 Bax/Bcl2 ratio of KKU-M213B cells

**ANOVA**

| | Sum of Squares | df | Mean Square | F | Sig. |
|---|---|---|---|---|---|
| Between Groups | 45.501 | 3 | 15.167 | 18.833 | .008 |
| Within Groups | 3.221 | 4 | .805 | | |
| Total | 48.723 | 7 | | | |

Duncan[a]

| | | Subset for alpha = 0.05 | |
|---|---|---|---|
| factor | N | 1 | 2 |
| 2.00 | 2 | .8325 | |
| 1.00 | 2 | 1.0000 | |
| 3.00 | 2 | 1.1800 | |
| 4.00 | 2 | | 6.5045 |
| Sig. | | .721 | 1.000 |

Means for groups in homogeneous subsets are displayed.

a. Uses Harmonic Mean Sample Size = 2.000.
