# Supplementary material for: Ethanolic extract of Ya-nang (Tiliacora triandra) leaf powder induces apoptosis in cholangiocarcinoma cell lines via induction of hyperacetylation and inhibition of growth signaling
Source: PeerJ. 2022 Dec 15;10:e14518. doi: 10.7717/peerj.14518 (PMC9760018; doi:10.7717/peerj.14518)
Supplement: Supplemental Information 4 — The films were cut, exposed, and developed manually, therefore, most small-size films were available as shown. [file peerj-10-14518-s004.pdf]

KKU-M213B cells (repeat 1)

Ac-H3 (17 kDa)

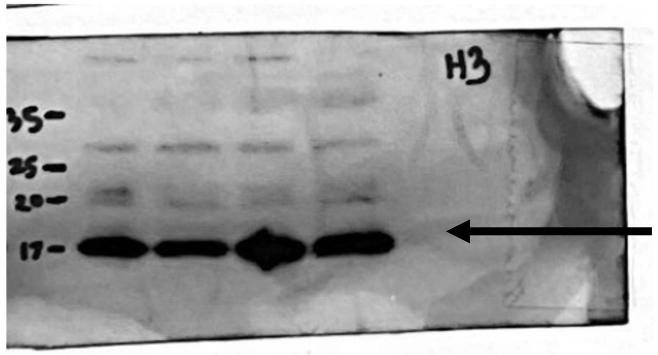

Ac-H3

C 31.25 62.5 125 TLPE (μg/mL)

P21 (21 kDa)

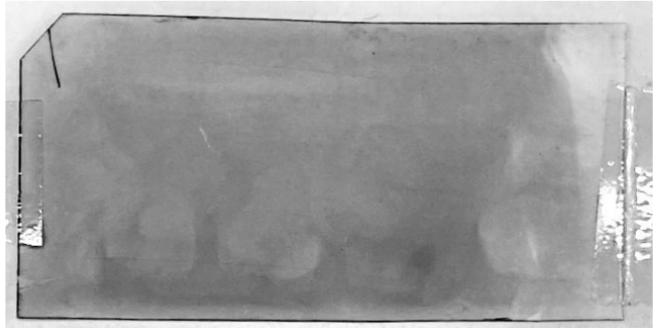

C 31.25 62.5 125 TLPE (μg/mL)

CDK4 (30 kDa)

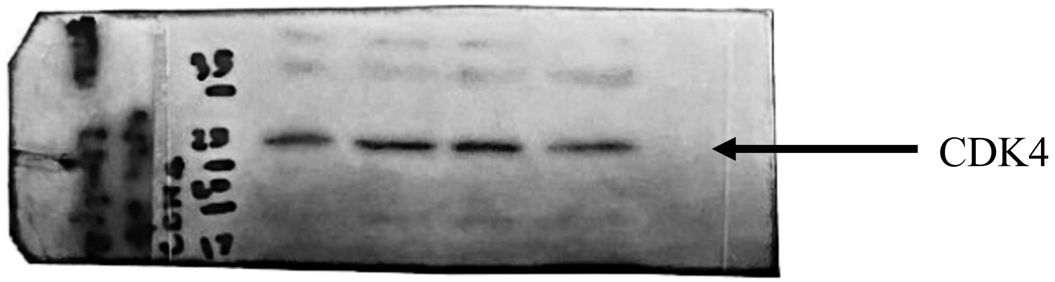

CDK4

C 31.25 62.5 125 TLPE (μg/mL)

p-ERK1/2 (42,44 kDa)

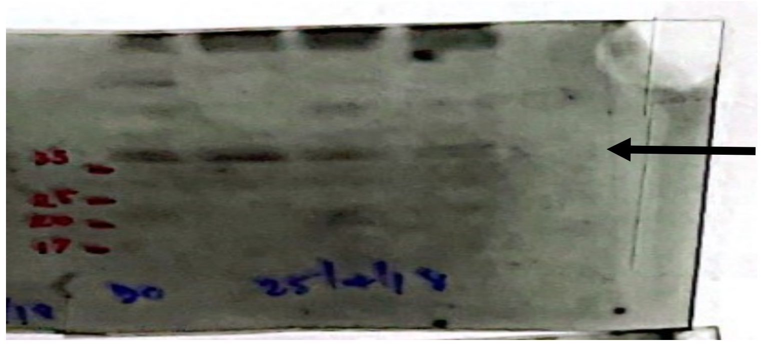

p-ERK1/2

C 31.25 62.5 125 TLPE (μg/mL)

P53 (53 kDa)

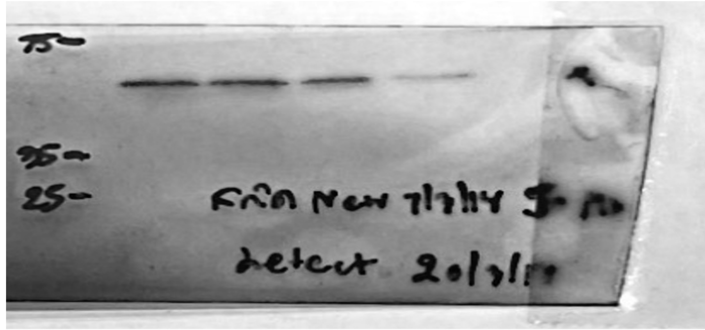

C 31.25 62.5 125 TLPE (μg/mL)

Bax (20 kDa)

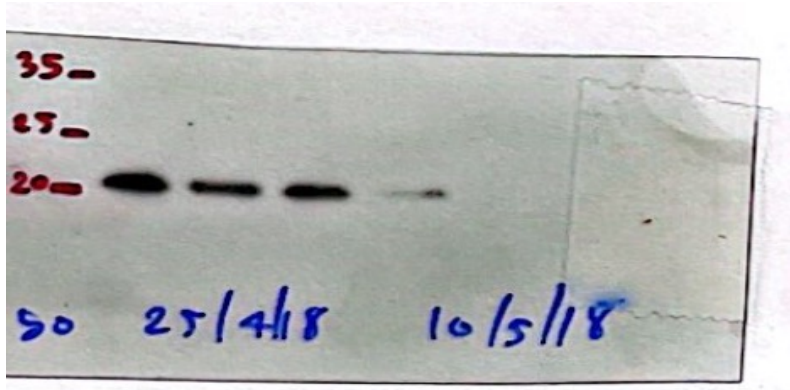

C 31.25 62.5 125 TLPE (μg/mL)

Total ERK1/2 (42,44 kDa)

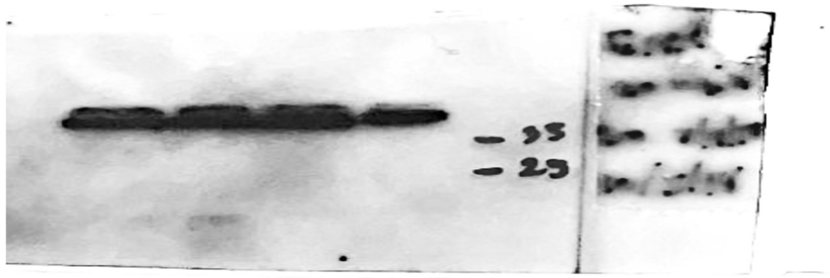

C 31.25 62.5 125 TLPE (μg/mL)

Bcl2 (26 kDa)

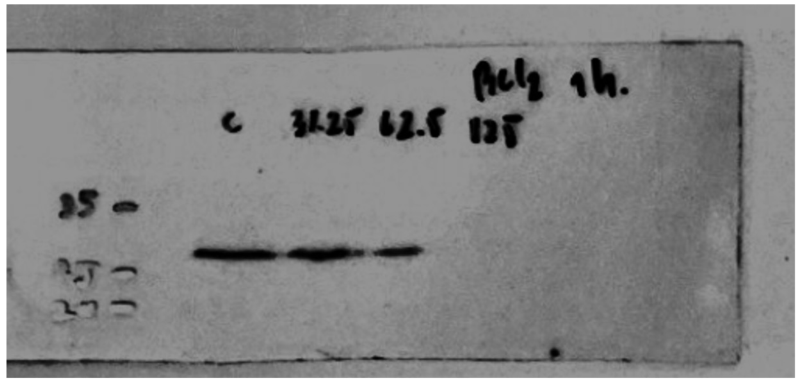

C 31.25 62.5 125 TLPE (μg/mL)

C= solvent control

KKU-M213B cells (repeat 2)

Ac-H3 (17 kDa)

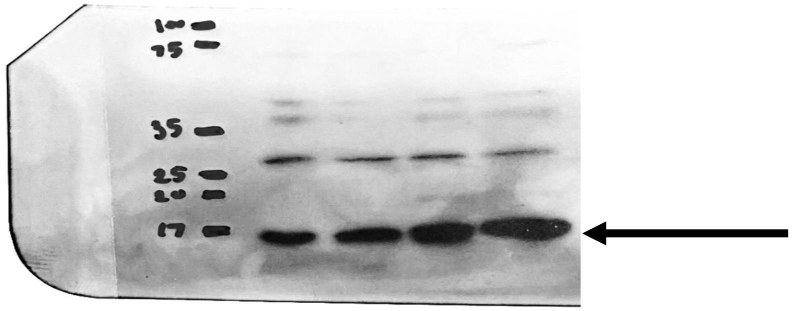

C 31.25 62.5 125 TLPE (µg/mL)

P21 (21 kDa)

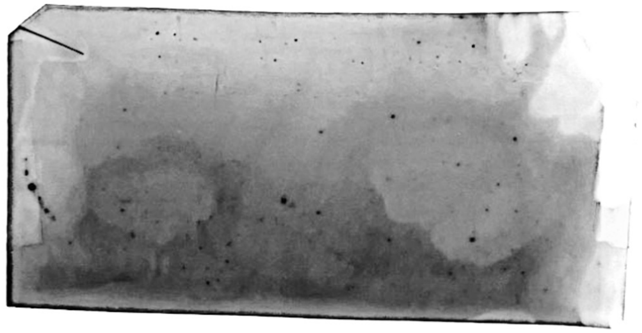

C 31.25 62.5 125 TLPE (µg/mL)

CDK4 (30 kDa)

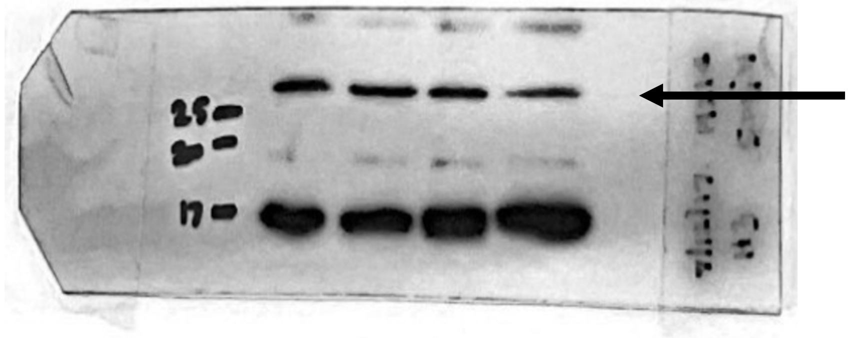

C 31.25 62.5 125 TLPE (µg/mL)

p-ERK1/2 (42,44 kDa)

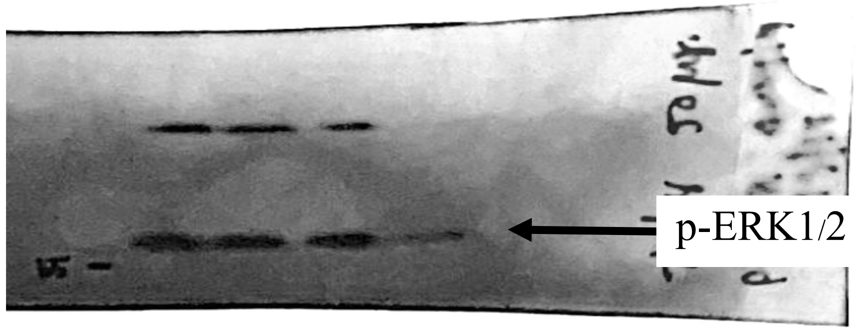

C 31.25 62.5 125 TLPE (µg/mL)

P53 (53 kDa)

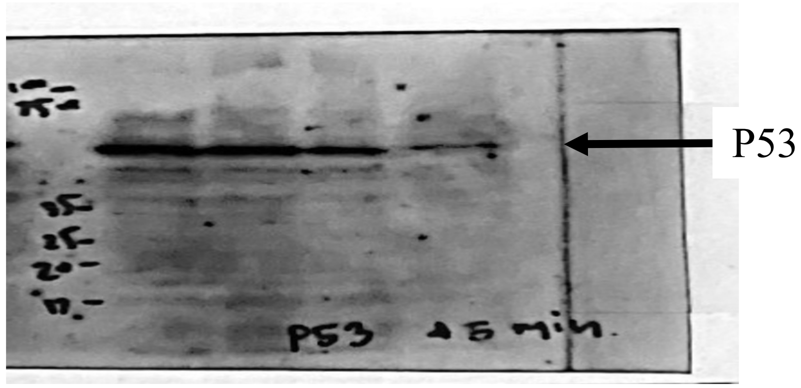

C 31.25 62.5 125 TLPE (µg/mL)

Bax (20 kDa)

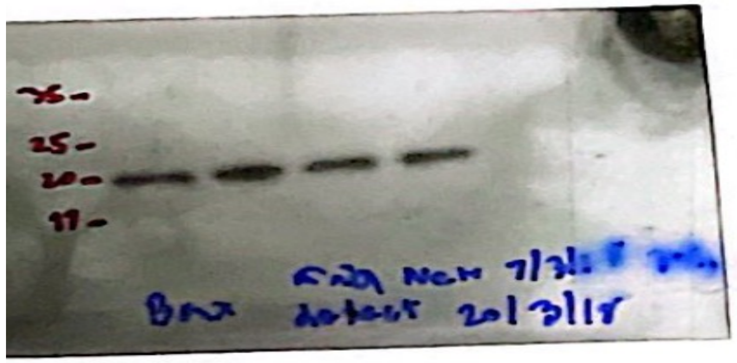

C 31.25 62.5 125 TLPE (µg/mL)

Total ERK1/2 (42,44 kDa)

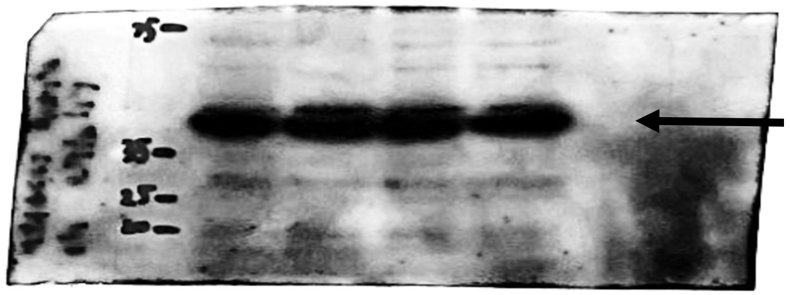

C 31.25 62.5 125 TLPE (µg/mL)

Bcl2 (26 kDa)

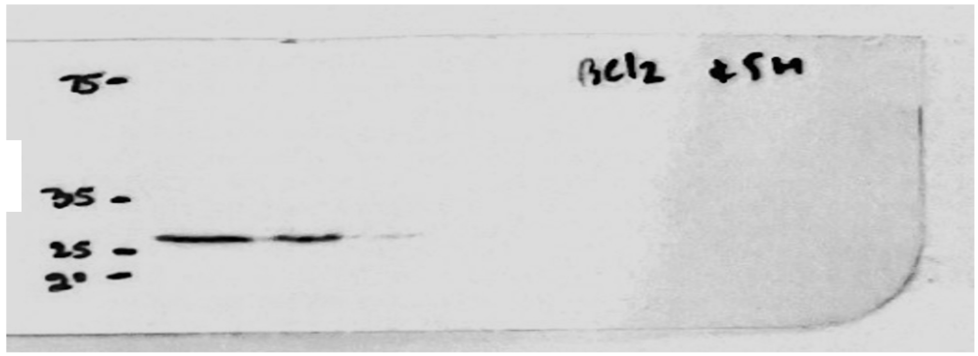

C 31.25 62.5 125 TLPE (µg/mL)

C= solvent control

KKU-100 cells (repeat 1)

Ac-H3 (17 kDa)

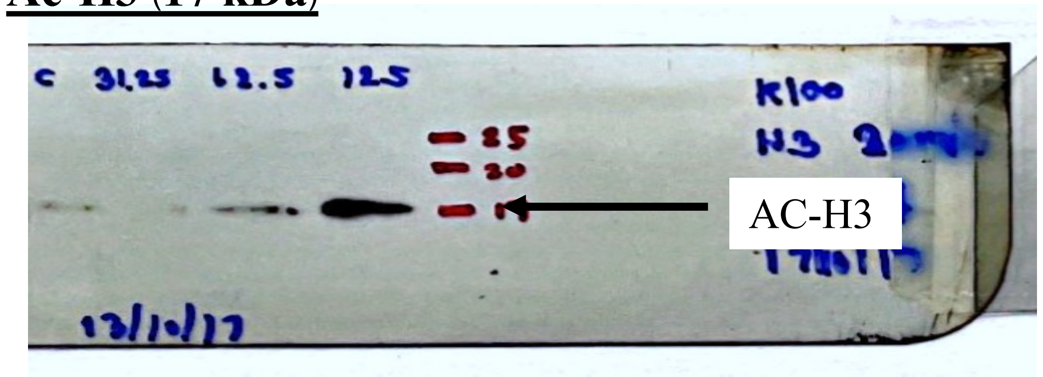

C 31.25 62.5 125 TLPE (µg/mL)

P21 (21 kDa)

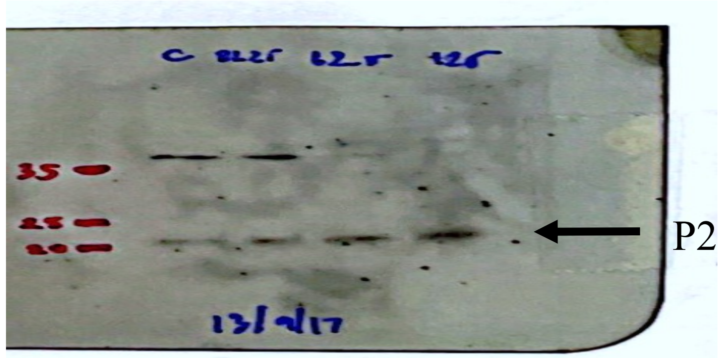

C 31.25 62.5 125 TLPE (µg/mL)

CDK4 (30 kDa)

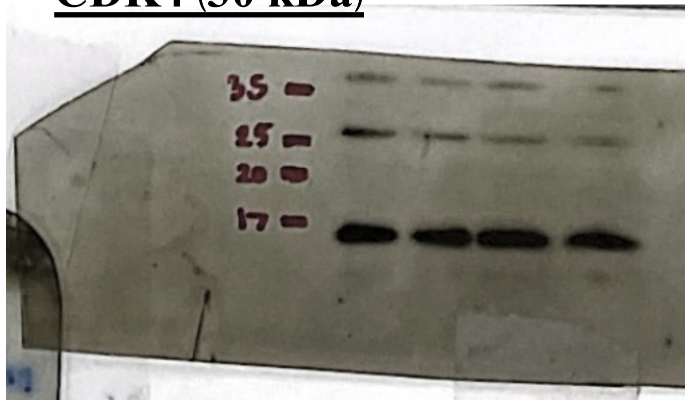

CDK4

C 31.25 62.5 125 TLPE (µg/mL)

p-ERK1/2 (42,44 kDa)

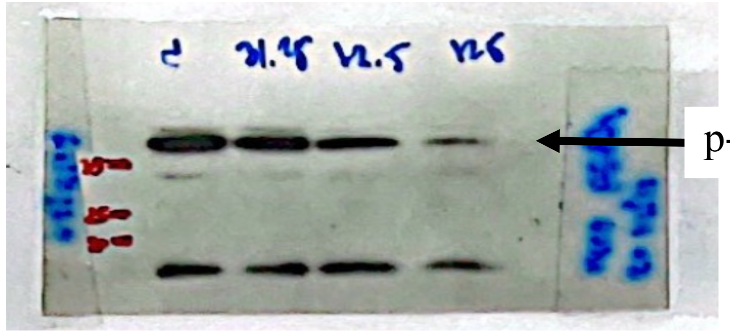

p-ERK1/2

C 31.25 62.5 125 TLPE (µg/mL)

P53 (53 kDa)

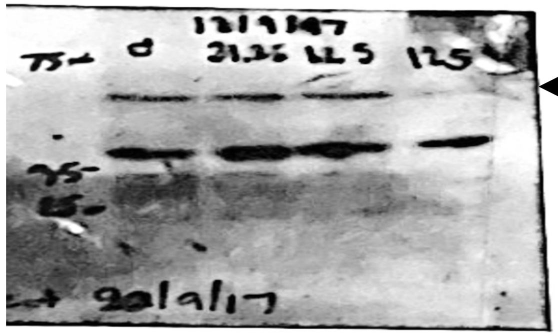

P53

C 31.25 62.5 125 TLPE (µg/mL)

Bax (20 kDa)

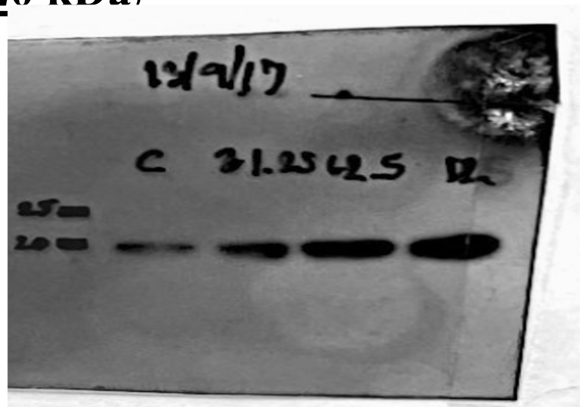

C 31.25 62.5 125 TLPE (µg/mL)

Total ERK1/2 (42,44 kDa)

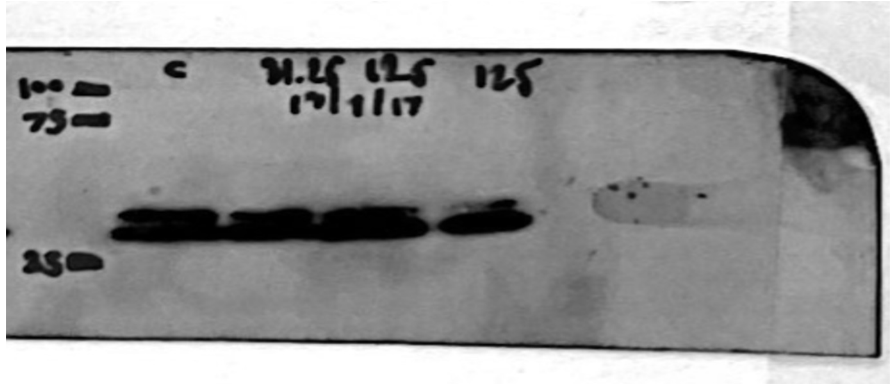

C 31.25 62.5 125 TLPE (µg/mL)

Bcl2 (26 kDa)

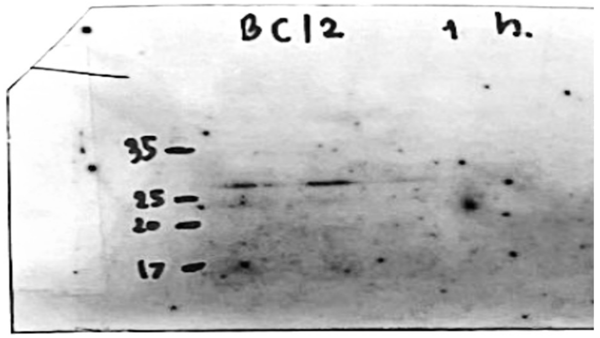

C 31.25 62.5 125 TLPE (µg/mL)

C= solvent control

KKU-100 cells (repeat 2)

Ac-H3 (17 kDa)

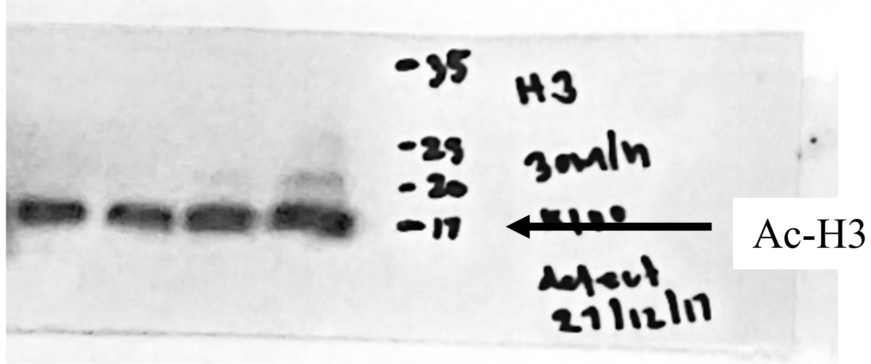

C 31.25 62.5 125 TLPE (µg/mL)

P21 (21 kDa)

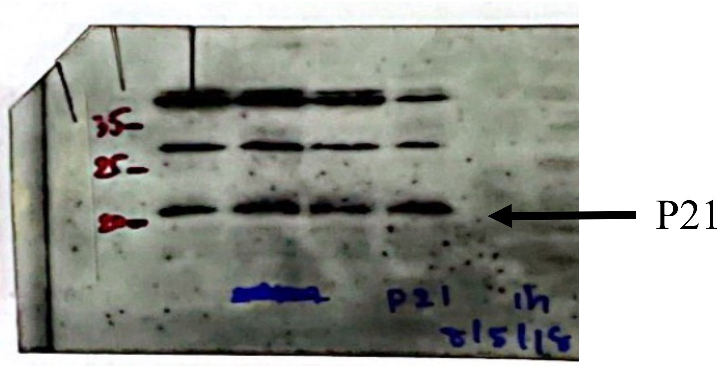

C 31.25 62.5 125 TLPE (µg/mL)

CDK4 (30 kDa)

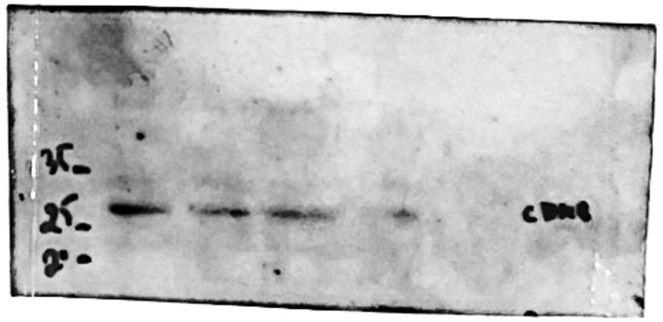

C 31.25 62.5 125 TLPE (µg/mL)

p-ERK1/2 (42,44 kDa)

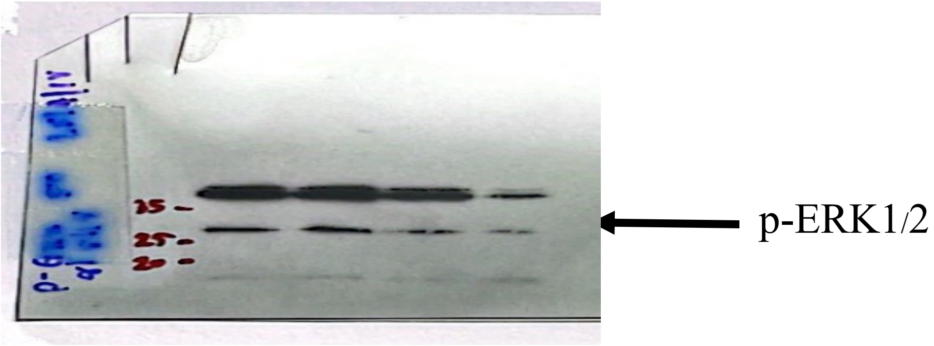

C 31.25 62.5 125 TLPE (µg/mL)

P53 (53 kDa)

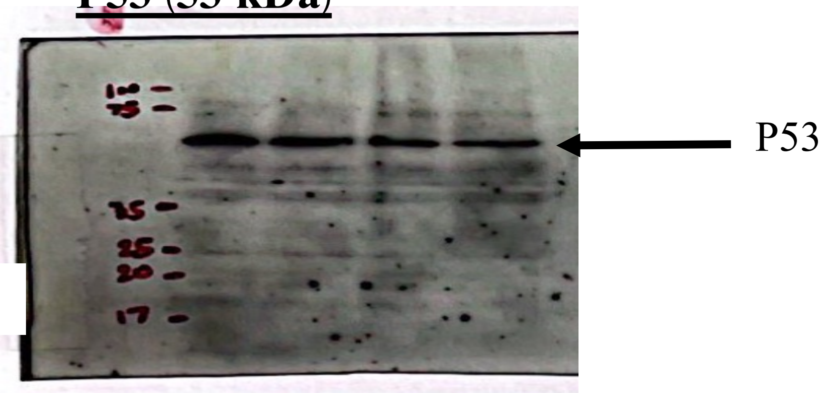

C 31.25 62.5 125 TLPE (µg/mL)

Bax (20 kDa)

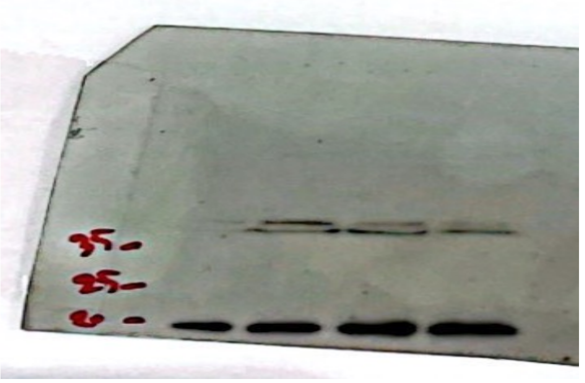

C 31.25 62.5 125 TLPE (µg/mL)

Total ERK1/2 (42,44 kDa)

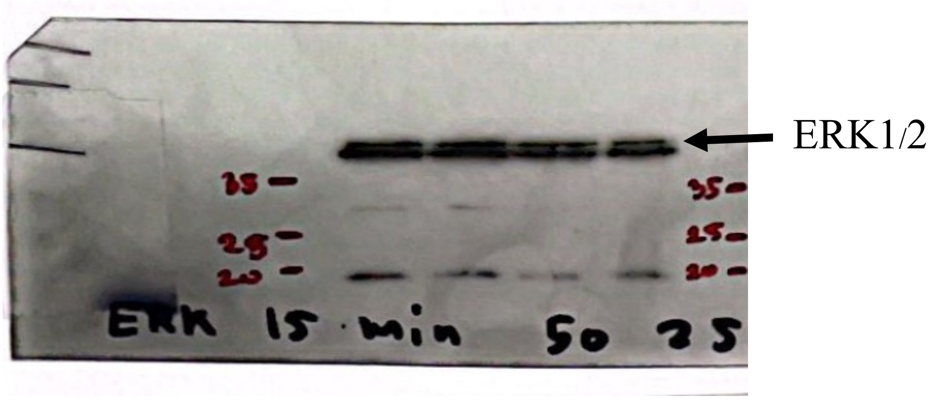

C 31.25 62.5 125 TLPE (µg/mL)

Bcl2 (26 kDa)

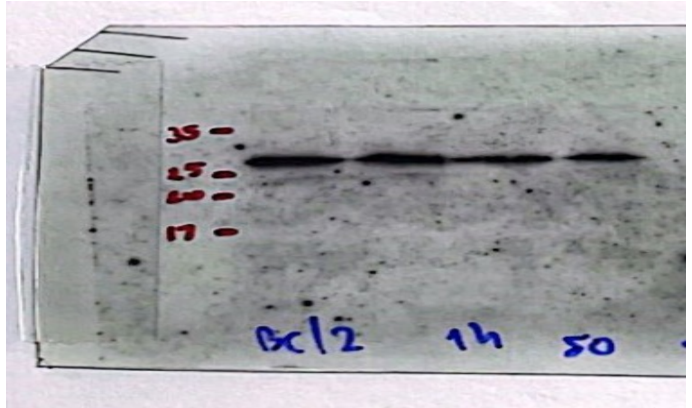

C 31.25 62.5 125 TLPE (µg/mL)

C= solvent control

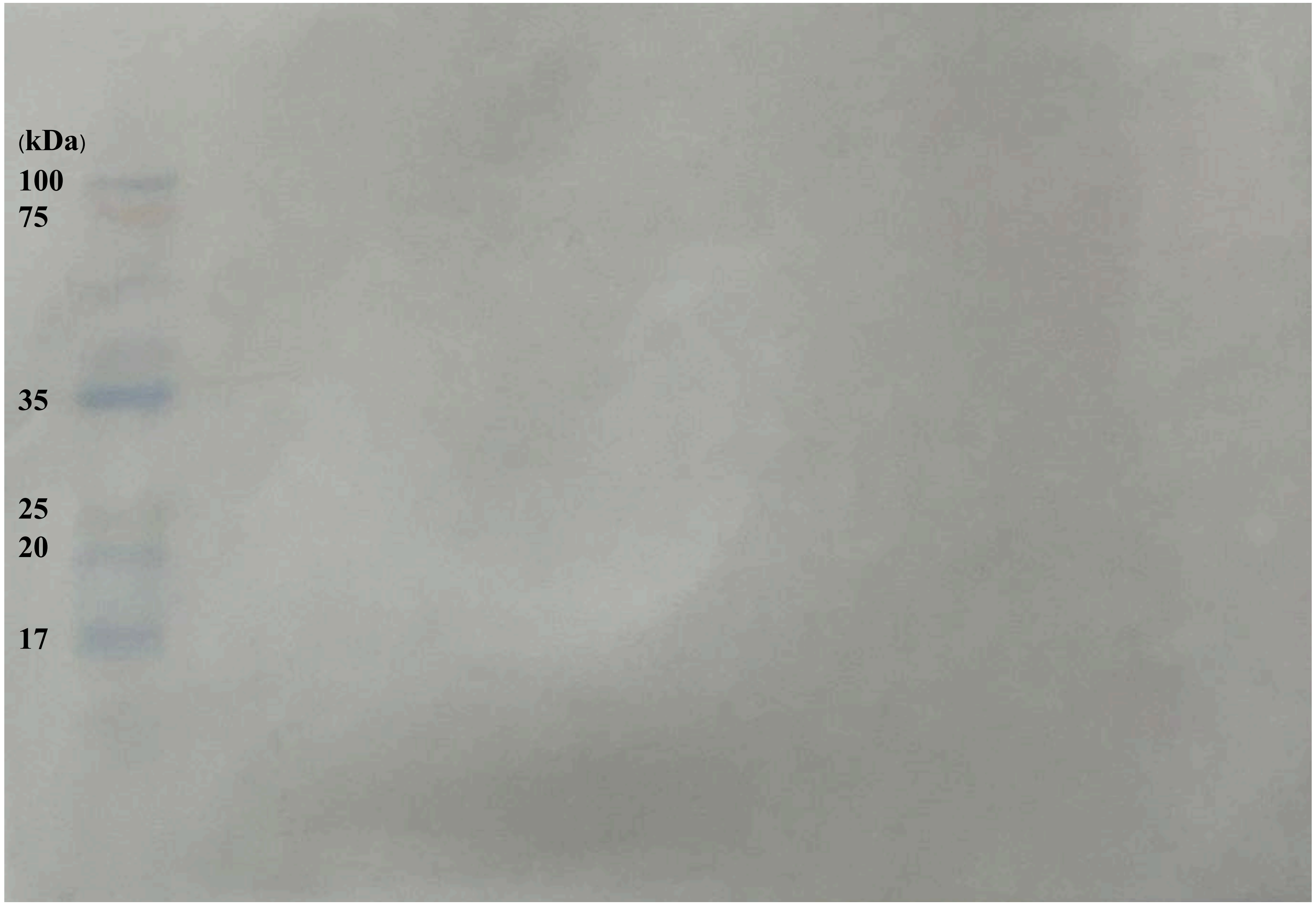

GeneDirex® BLeye Prestained Protein Ladder on PVDF membrane
